# Chemical Constituents from *Osmanthus fragrans* var. *aurantiacus* Makino with Their In Vitro and In Silico Studies Target Anti-Inflammation by Suppressing ERK 1/2 MAPK Signaling

**DOI:** 10.3390/ijms26178421

**Published:** 2025-08-29

**Authors:** Ducdat Le, Thinhulinh Dang, Vinhquang Truong, Thientam Dinh, Soojung Yu, Seok-Geun Lee, Mina Lee

**Affiliations:** 1 College of Pharmacy and Research Institute of Life and Pharmaceutical Sciences, Sunchon National University, 255 Jungangno, Suncheon 57922, Republic of Korea; ddle@scnu.ac.kr (D.L.);; 2 Department of Natural Cosmetics Science and Smart Beautytech Research Institute, Sunchon National University, 255 Jungangno, Suncheon 57922, Republic of Korea; 3 Graduate School, Kyung Hee University, Seoul 02447, Republic of Korea; seokgeun@khu.ac.kr; 4 BioNanocomposite Research Center, Kyung Hee University, Seoul 02447, Republic of Korea

**Keywords:** *Osmanthus fragrans*, isolated compounds, inflammation, antioxidant, ERK 1/2 MAPK

## Abstract

*Osmanthus fragrans* var. *aurantiacus* Makino is a traditional medicine for treating various diseases, including inflammation. In this study, we discovered the biological features of this plant by assessing antioxidative and anti-inflammatory activities. The GNPS-FBMN approach and in vitro assays guided the identification of active ingredients. As a result, one new compound and 17 other compounds were separated and identified. The structure of the new compound was established by CD spectrum and hydrolysis, followed by HPLC analysis. These compounds demonstrated antioxidative and anti-inflammatory activities. Western blotting clarified the active compound by inhibiting inflammation through COX-2 and iNOS enzymes and blocking the ERK 1/2 MAPK signaling. In silico approaches supported the binding affinity and dynamic features of the established complexes’ target inflammation. Our finding supports evidence from both experimental and in silico approaches that *O. fragrans* fractions and its constituents may be employed as potential therapeutic phytochemicals for treating inflammatory bowel diseases.

## 1. Introduction

Inflammation is a basic biological reaction to injury stimuli. It plays a role in the pathophysiology of many chronic illnesses, such as cancer, inflammatory bowel disease (IBD), and rheumatoid arthritis, after dysregulating [1]. Nitric oxide (NO) is a flexible, short-lived gaseous molecule that has a dual role in physiological and pathological processes. It is one of the major molecular mediators involved in the inflammatory cascade. NO is crucial for controlling neurotransmission, host defense, and vascular tone under homeostatic circumstances [2]. However, during inflammation, its synthesis is significantly dysregulated, which plays a role in the pathophysiology of numerous chronic inflammatory diseases, such as asthma, IBD, rheumatoid arthritis, and neurodegenerative diseases. These molecules are crucial for managing blood vessel function, organizing immune responses, and encouraging the production of substances that cause inflammation [3].

*Osmanthus fragrans* var. *aurantiacus* Makino is a native Asian perennial plant. This plant is either scented with blossoms or has extensive folk remedies. Traditionally, several parts of the plant have been used to treat a wide variety of diseases, such as inflammation, skin disorders, digestive problems, and coughs. Previous studies of this plant indicated a broad diversity of phytochemical components, such as phenolics [4], triterpenoids [5], volatiles and odor actives [6], and other compounds [7].

Among the active constituents, triterpenoids and phenolics extracted from the leaves have shown substantial anti-inflammatory activities, suppressing pro-inflammatory cytokines and pathways, such as cyclooxygenase-2 (COX-2) and nuclear factor kappa B (NF-κB) [8]. Phillyrin was identified from this plant, having multiple bioactivities such as antioxidants, anti-tumor, anti-inflammatory, antiviral, antibacterial, and weight loss. This compound also displayed some pharmacological effects, involving a variety of potential signaling pathways, such as nuclear factor erythroid 2-related factor 2, nuclear factor kappa B, toll-like receptor, phosphoinositide 3-kinase/protein kinase B, and mechanisms related to polycyclic polyprenylated acylphloroglucinols [5]. Furthermore, additional active ingredients in *O. fragrans* also showed encouraging suppression of b-site amyloid precursor protein cleaving enzyme 1 activity, suggesting that they may be used to treat Alzheimer’s disease [4]. Additionally, the ethyl acetate fraction of *O. fragrans* effectively suppresses cell proliferation and survival in colorectal cancer cells, attributing these effects to the inhibition of COX-2 and NF-κB pathways [8]. The interactions between several phytochemicals in *O. fragrans* suggest a synergistic effect that can reinforce its therapeutic applications in traditional and modern medicine [6]. As research progresses, a detailed understanding of its mechanisms can lead to new therapeutic strategies that take advantage of the anti-inflammatory capacities of these phytochemicals, ultimately contributing to better health results [7,9]. The ethopharmacological uses of *O. fragrans* for the management of inflammation suggested the underlying mechanisms, and the bioactive components of *O. fragrans* present a promising border in the fight against inflammatory diseases.

In recent years, a number of research groups have used the Global Natural Products Social Molecular Networking (GNPS) platform to transform natural product discovery into a community-driven and accessible process for all scientists investigating small molecules. The rich structures and variety of bioactivities bestowed to natural products have been recognized for a long time and are well recognized as important sources for drug discovery and development [10]. GNPS-FBMN addresses these challenges through the integration of mass spectrometry-based molecular networking with fragmentation-based similarity searching, enabling researchers to rapidly annotate and prioritize natural products based on their fragmentation patterns and matching scores. Molecules that exhibit similar fragmentation patterns are grouped together into networks, with each node representing a distinct molecule and the edges connecting nodes representing the degree of similarity between their fragmentation spectra. This approach allows for the visualization of complex datasets and the identification of molecular structures [11].

Continuing our efforts to discover the active constituents for treating inflammatory diseases, our primary study suggests that the extract and fractions of *O. fragrans* have potent anti-inflammatory capacity. Thus, this study aims to discover the chemical constituents from the active fractions of *O*. *fragrans’* targeted anti-inflammatory effects. Eighteen compounds were identified and evaluated for their anti-inflammatory effect by using both experimental and in silico approaches targeting the ERK 1/2 MAPK pathway to clarify the anti-inflammatory properties of this plant.

## 2. Results

### 2.1. Bioactivities Guided Isolation of Constituents from O. fragrans Leaves

**Figure 1:**
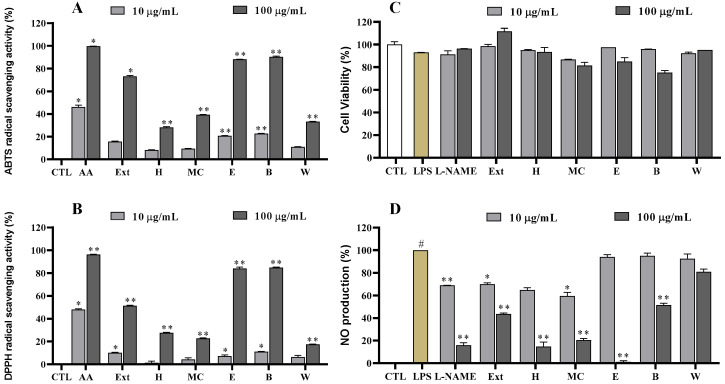
Antioxidative and anti-inflammatory effects of the total extract (Ext) and (H, MC, E, B, and W) fractions of *O. fragrans* leaves. (**A**) ABTS and (**B**) DPPH radical-scavenging activity. Negative control (CTL, White bar) was prepared by replacing sample with the same volume of solvent. Ascorbic acid was used as a positive control (AA). RAW264.7 cells were added with samples at different concentrations. After 1 h, cells were further stimulated with LPS (1 µg/mL) for 16 h. (**C**) Cell viability was assessed by using MTT assay. (**D**) NO production was measured at 550 nm using a microplate reader. L-NG-Nitro arginine methyl ester (L-NAME) served as a positive control. Samples concentrations were tested at 10 and 100 μg/mL. Each experiment was conducted three times. The data are expressed as mean ± SD. * *p* < 0.05, ** *p* < 0.01 vs. LPS-treated group (yellow bar). ^#^ *p* < 0.01 vs. control.

To examine the antioxidative effects of this plant, we screen its extract (Ext) and fractions [*n*-hexane (H), dichloromethane (MC), ethyl acetate (E), butanol (B), and distilled water (W)] by using DPPH and ABTS assays to discover their ability to scavenge radicals (Figure 1A,B). In both assays, the control showed a low scavenging activity at all tested concentrations, suggesting that there is no antioxidant content. Ascorbic acid (AA) served as the positive control for both assays, exhibiting potent radical-scavenging activities at both tested concentrations of 10 and 100 µg/mL. For the ABTS radical (Figure 1A), the extract and fractions display varying antioxidative effects. All tested samples demonstrated antioxidative effects at both concentrations three times. At 100 µg/mL, the extract, E, and B fractions exhibited potent, strong radical-scavenging activity with rates of 73.1 ± 15.6%, 88.3 ± 20.84%, and 90.3 ± 22.7%, respectively. MC, W, and H fractions showed some radical-scavenging activity at 39.3 ± 9.4%, 33.3 ± 10.9%, and 28.1 ± 8.23%, respectively. Samples displayed radical-scavenging activity at 10 µg/mL. Therefore, these samples potentially scavenged ABTS radicals by donating electrons or hydrogen atoms to neutralize ABTS radicals, resulting in a reduction in its absorbance and a color change from blue–green to colorless. For the DPPH assay (Figure 1B), a similar effect was observed for the radical-scavenging activity of these samples. At 100 µg/mL, all samples exhibited an antioxidative effect. The B and E fractions exhibited strong radical-scavenging activity, with rates of 84.1 ± 7.1% and 84.8 ± 11.0%, respectively, compared to the control. The extract also scavenged the DPPH radical with a rate of 51.3 ± 10.5% compared to that of the control. The other fractions exhibited scavenging activity with rates of 27.5–17.4%. Except for the H fraction, all samples displayed weak radical-scavenging activity at 10 µg/mL (Figure 1B). It is notable that the radical-scavenging activity of these samples was dose-dependently regulated by increasing efficiency scale with concentration. It is proposed that active samples combat oxidative stress by neutralizing free radicals.

To investigate the anti-inflammatory effects of the extract and fraction, we employed a NO assay to determine LPS-generated NO production (*p* < 0.01) in RAW264.7 cells in the presence of samples compared to those of the LPS treatment without sample addition. At first, all samples were evaluated for their effectiveness for cell viability (Figure 1C). Except for fraction B, these samples did not cause significant toxicity to RAW264.7 cells. The control bar did not show NO production due to the absence of LPS activation in RAW264.7 cells. After stimulation with LPS, the LPS elicits NO production activated in RAW264.7 cells, indicating a strong inflammatory stimulation. L-NAME (positive control) strongly inhibited NO production compared to the LPS bar, confirming its inhibitory effect on NO production. By treating the extract and fractions, the NO production was altered in a different manner. At 100 µg/mL, the E fraction showed the strongest inhibition and significant suppression (*p* < 0.01) of NO production in LPS-activated RAW264.7 cells by inhibiting 99.7 ± 2.3% of NO production compared to that of the LPS bar. H and MC fractions also inhibited NO production with high inhibition rates of 85.4 ± 4.05 and 79.7 ± 1.5%, respectively, which are similar to those of the positive control (84.1 ± 2.3%), compared to those of the LPS bar without sample addition. The extract and B fraction showed moderate inhibition with rates of 56.5 ± 0.92% and 48.6 ± 1.62%, respectively, compared to the LPS bar. The W fraction displayed a weak inhibition (Figure 1D) compared to the LPS bar. Error bars (mean ± SD) indicated low variability among replicates, supporting the reproducibility of the observed inhibitory effects. These results suggest that the extract of *O. fragrans* leaves could be employed for further experiments

### 2.2. Investigation of Chemical Constituents Using GNPS-FBMN Approach

**Figure 2:**
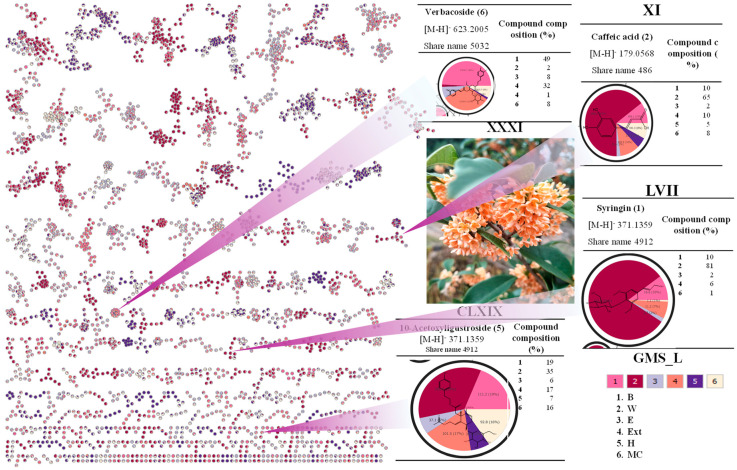
GNPS-FBMN approach to extract (Ext: orange) and fractions [B: pink; W: red; E: light purple; H: violet; and MC: light yellow] of *O. fragrans*.

The chemical composition of secondary metabolites from *O. fragrans* was discovered by using a GNPS-FBMN (Global Natural Products Social Molecular Networking–Feature-Based Molecular Networking) approach. Isotopic pattern analysis of experimental MS/MS data was integrated with spectral matching against the GNPS open platform. The identification of compounds was achieved through high cosine score matches (threshold > 0.7) with reference spectra. The predicted compounds were achieved based on high cosine score matches to reference spectra. These annotations were based on mass spectral dereplication and authentication via high spectral matching. Putative compound annotations were further refined through isotope pattern similarity from clustering (Figure 2). This approach allowed for the prediction and identification of compounds within *O. fragrans* extracts and fractions. As illustrated in Figure 2, the FBMN analysis annotated known compounds, including phenolic derivatives such as syringin (**1**), caffeic acid (**2**), 10-acetoxyligustroside (**5**), and verbacoside (**6**). Among them, compounds **2**, **5**, and **6** have been previously reported in *O. fragrans* [6].

**Scheme 1:**
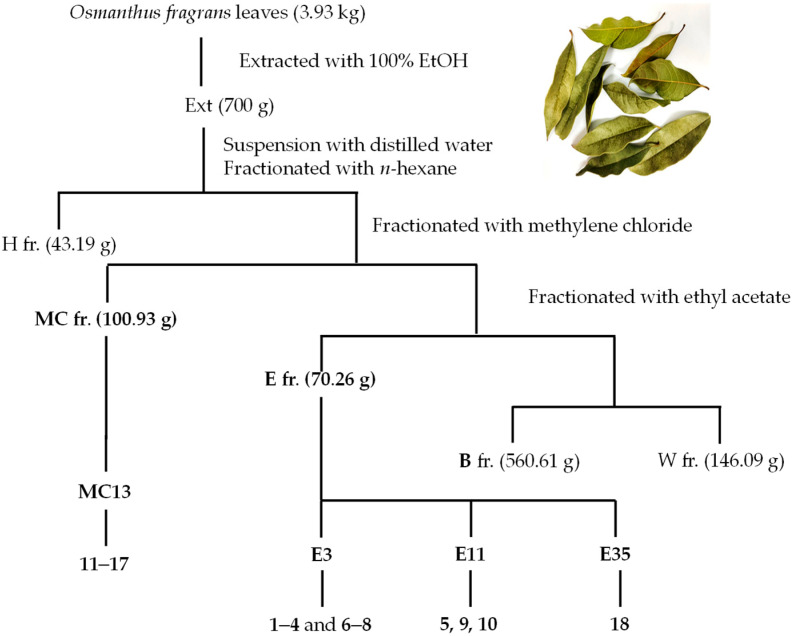
Extraction and separation of compounds (**1**–**18**).

To separate the active constituents (Scheme 1) from the active fractions, E and MC fractions were further separated by using multiple chromatographic conditions to achieve 18 compounds. Their structures were determined by an analysis of their spectroscopic and mass data in comparison to those reported references.

**Figure 3:**
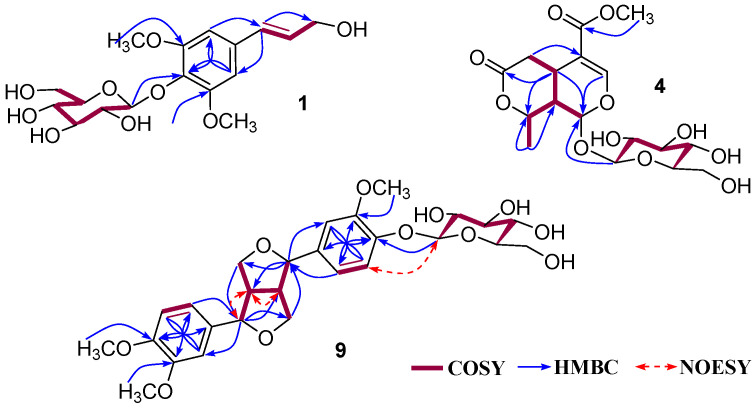
Key 2D NMR corrections of compounds **1**, **4,** and **9**.

The high-resolution mass spectrum (HRESIMS) of compound **1** showed an ammonium adduct ion peak at *m*/*z* 390.1740 [M + NH_4_]^+^ (*calcd*. for C_17_H_24_O_9_NH_4_, 390.1764). At the same time, some fragments were also observed at *m*/*z* 193.0865, suggesting a reduction of an *O*-Glc moiety. On the other hand, other fragments at *m*/*z* 180.0858 and 161.0589 were also observed, revealing the presence of its glucose unit [12]. The ^1^H NMR spectrum of **1** revealed the singlet signals of a meta-coupling system at *δ*_H_ 6.75 (brs, H-2, 6), a *trans*-olefinic system at *δ*_H_ 6.55 (d, *J* = 15.8 Hz, H-7) and 6.33 (dt, *J* = 15.8, 5.6 Hz, H-8), and other oxygenated protons around 4.22–3.20 (Table 1). Especially, an anomeric proton at *δ*_H_ 4.87 (d, *J* = 7.7 Hz, H-1′) was observed, suggesting a *β*-D sugar unit. The ^13^C NMR spectrum of **1** displayed 15 signals, including three oxygenated carbons at *δ*_C_ 154.4 (C-3, 5) and 135.9 (C-4), four unsaturated carbons at *δ*_C_ 131.3 (C-7), 130.1 (C-8), and 105.5 (C-2, 6), and an oxygenated methylene at *δ*_C_ 63.0 (C-9), together with six carbons of sugar unit (*δ*_C_ 103.4, 77.9 × 2, 75.8, 71.4, and 62.6) (Table 1). The COSY spectrum showed a sequence correlation of H-7/H-8/H2-9, assigned to the side chain of **1**. The HMBC spectrum showed the correlation of an anomeric proton at H-1′ (*δ*_H_ 4.87) to C-4 (*δ*_C_ 135.9) and those of H-2/H-6 (*δ*_H_ 6.75) to C-4 (*δ*_C_ 135.9)/C-7 (*δ*_C_ 131.3) (Figure 3), suggesting for assignment of 4-*O*-glc and the side chain attached to C-1, respectively. The spectroscopic data (Figures S1–S6, Supporting Materials) of **1** was identical to those of syringin [13].

The HRESIMS spectrum of **4** displayed a protonated ion peak at *m*/*z* 405.1368 [M + H]^+^ (*calcd*. for C_17_H_25_O_11_, 405.1397). At the same time, a fragment at *m*/*z* 243.0849 was observed for [M + H − Glc]^+^, suggesting a reduction of a glc moiety. On the other hand, the fragmentation pathway of **4** showed ion peaks at *m*/*z* 228.2310 and 211.0588, revealed for [M + H − Glc − CH_3_]^+^ and [M + H − Glc − CH_3_ − H_2_O]^+^, respectively. The ^1^H NMR spectrum of **4** showed a methoxycarbonyl singlet at *δ*_H_ 3.73 (11-*O*CH_3_), an olefinic proton at *δ*_H_ 7.58 (brs, H-3), and an anomeric proton for the *β*-conformer of a sugar at *δ*_H_ 4.70 (d, *J* = 7.9 Hz, H-1′), characterized by a secoiridoid glycoside. The large coupling constants of H-1 (*δ*_H_ 5.50, d, *J* = 7.9 Hz) and CH_3_-10 (*δ*_H_ 1.51, d, *J* = 6.4 Hz) were suggested for C-1*S* and C-8*R* conformers, respectively [12]. The ^13^C NMR spectrum of **4** displayed 17 signals, including 11 carbons of the secoiridoid backbone and six carbons of the sugar unit (Table 1). The COSY spectrum showed a sequence correlation of H-1/H-9/H-8/H-5, and those between H-8 and H-10, and between H-5 and H-6, further confirmed the above secoiridoid structure. Especially, the HMBC spectrum displayed a long-range correlation between CH_3_ (*δ*_H_ 3.73) and C-11 (*δ*_C_ 168.3) and those of H-1′ (*δ*_H_ 4.70) to C-1 (*δ*_C_ 96.3) (Figure 3), indicating CH_3_-11 and C-1-*O*-Glc attachments. The spectroscopic data (Figures S7–S12, Supporting Materials) of **4** was consistent with those of a previous report [14]. Therefore, compound **4** was identified as 8-epikingiside.

The HRESIMS spectrum of **9** displayed an ammonium adduct ion peak at *m*/*z* 552.2411 [M + NH_4_]^+^ (*calcd*. for C_27_H_34_O_11_NH_4_, 552.2445). At the same time, a fragment at *m*/*z* 355.1522 was observed for [M + NH_4_ − *O*Glc]^+^, suggesting a reduction of a Glc moiety. The ^1^H NMR spectrum of **9** revealed two ABX spin coupling systems at *δ*_H_ 7.15 (d, *J* = 8.3 Hz, H-5), 7.03 (d, *J* = 2.0 Hz, H-2), 6.93 (dd, *J* = 8.3, 2.0 Hz, H-6), and 7.01 (d, *J* = 1.8 Hz, H-2′), 6.94 (m, H-5′, 6′), two benzylic oxymethine at *δ*_H_ 4.89 (d, *J* = 5.9 Hz, H-7′) and 4.48 (d, *J* = 6.8 Hz, H-7), two oxymethylene groups, two methines *δ*_H_ 2.94 (m, H-8), 3.41 (m, H-8′), an anomeric signal at *δ*_H_ 4.88 (d, *J* = 7.4 Hz, H-1′′), other oxygenated protons around 3.67–3.38 ppm, and three oxymethyl groups at *δ*_H_ 3.87, 3.85, and 3.83 (s, for each) (Table 1) (Figure S13, Supporting Materials). The ^13^C NMR spectrum of **9** displayed 27 carbons, including two benzoyl moieties, a tetrahydrofurofuran, three methoxy groups, and a sugar unit. The spectroscopic data of **9** proposed a structure of a tetrahydrofurofuran ligand glycoside [14] (Figure S14, Supporting Materials). The COSY spectrum showed a sequence correlation of H-7/H-8/H-9 and those of H-7′/H-8′/H-9′, confirming the substructure of the tetrahydrofurofuran unit. The HMBC spectrum showed the long-range correlations of H-2/H-6 to C-7 and those of H-2′/H-6′ to C-7′, suggesting the establishment of 1,3,4-trisubstituted benzoyl tetrahydrofurofuran aglycone. The HMBC long-range correlation between H-1′′ (*δ*_H_ 4.88) and C-4 (*δ*_C_ 147.6), together with a NOESY cross-peak between H-1′′ (*δ*_H_ 4.88) and H-5 (*δ*_H_ 7.15), confirmed C-4-*O*-Glc attachment. The HMBC spectrum also demonstrated the correlations of CH_3_ (*δ*_H_ 3.87, 3.85, and 3.83) to C-3, C-3′, and C-4′, respectively, indicating methylation at C-3, C-3′, and C-4′ (Figure 3). The relative configuration of the 8-H/8′-H type furofuran moiety was primarily determined by analyzing the chemical shift differences of H_2_-9 (Δ*δ*_H_-9) and H_2_-9′ (Δ*δ*_H_-9′) [15]. Indeed, the small value of (Δ*δ*_H_-9 = 0.27) and a large value (Δ*δ*_H_-9′ = 0.52) (Table 1) suggested for H-7/H-8 and H-7′/H-8′ as *trans*- and *cis*-configurations, respectively. The large coupling constant (*J*_7,8_ = 6.8 Hz) suggested that two protons are located on opposite sides of the structural plane. Especially, the NOESY spectrum showed significant correlations between H-7′ and H-8′, revealing that they are located on the same sides of the structural plane (Figures S15–S20, Supporting Materials). Additionally, the NOESY correlation between H-8 and H-8′ indicated their equatorial positions. The absolute configuration of the 8-H/8′-H type furofuran moiety was finally confirmed by the positive Cotton effect around 242 nm and negative Cotton effect around 288 nm in the CD spectrum (Figure S21, Supporting Materials), indicating a 7*S*, 8*R*, 7′*R*, 8′*R* configuration [16]. The absolute configuration of the sugar moiety was determined as *β*-d glucose by acid hydrolysis and HPLC analysis using our previous method [12]. Accordingly, **9** was identified as a new compound and named fragrans D1.

**Figure 4:**
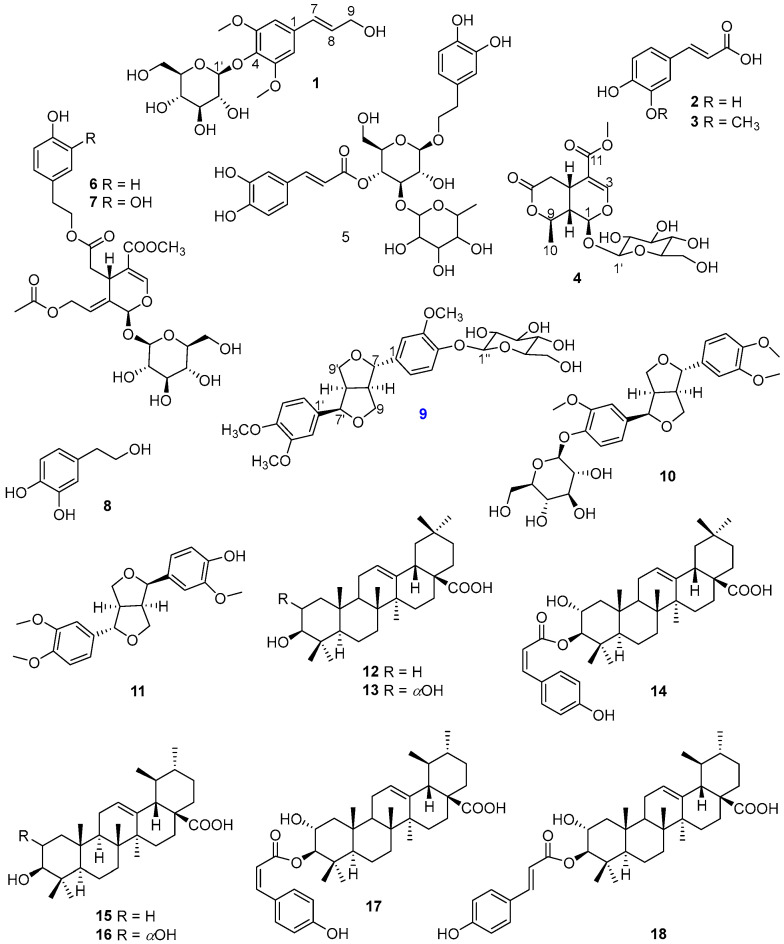
Chemical structures of isolated compounds from *O. fragrans* leaves.

Furthermore, the structures of the additional 15 compounds (Figure 4) were identified by an analysis of their spectroscopic data and compared to those reported in the literature. Their structures were elucidated as caffeic acid (**2**) [17], ferulic acid (**3**) [18], 10-acetoxyligustroside (**5**) [19], verbacoside (**6**) [20], 10-acetoxyoleuropein (**7**) [21], hydroxytyrosol (**8**) [22], phillyrin (**10**) [22], (+)-phillygenin (**11**) [23], oleanonic acid (**12**), maslinic acid (**13**) [24], 3-*O*-*cis*-coumaroylmaslinic acid (**14**) [25], ursolic acid (**15**) [26], corosolic acid (**16**) [27], 3*β*-*cis*-*p*-coumaroyloxy-2*α*-hydroxyl-urs-12-en-28-oic acid (**17**), and 3*β*-*trans-p*-coumaroyloxy-2α-hydroxyl-urs-12-en-28-oic acid (**18**) [28].

### 2.3. Antioxidative Effects of Compounds *(**1**–**18**)*

**Figure 5:**
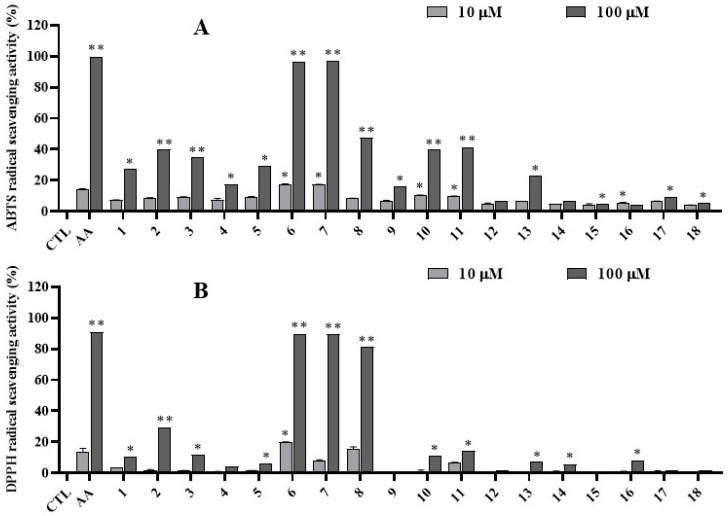
Antioxidative effect of isolated compounds (**1**–**18**) isolated from *O. fragrans* leaves by scavenging ABTS (**A**) and DPPH (**B**) radicals. Sample concentrations were tested at 10 and 100 μM. Each experiment was conducted in triplicate. The data are expressed as mean ± SD. * *p* < 0.05, ** *p* < 0.01 vs. control.

These isolated compounds (**1**–**18**) were tested for antioxidative effect by scavenging ABTS and DPPH radicals (Figure 5). The control bar did not display potent radical-scavenging activity due to a lack of antioxidants. For ABTS radicals at 100 µM, compounds **6** and **7** demonstrated strong radical-scavenging activity, with rates of 96.3 ± 0.1% and 97.0 ± 0.6%, respectively, which are similar to those of AA (99.5 ± 0.1%). Compounds **2**, **3**, **8**, **10**, and **11** displayed moderate radical-scavenging activity, with rates ranging from 27.3 ± 0.1% to 47.5 ± 0.2%. Compounds **1**, **4**, **5**, and **13** showed some radical-scavenging activity, with rates around 22.9 ± 29.1%. At 10 µM, compounds **6** and **7** also displayed stronger activity than that of the positive control. Other compounds did not have a significant effect on radical-scavenging activity under experimental conditions (Figure 5A).

For DPPH radicals at 100 µM, compounds **6**–**8** displayed a comparative radical-scavenging activity, with rates of 89.8 ± 0.8%, 89.6 ± 0.1%, and 81.4 ± 2.1%, respectively, compared to the positive control (90.9 ± 0.4%). Compound **2** showed a moderate radical-scavenging activity, with a rate of 29.5 ± 1.3%. At 10 µM, compounds **6** and **8** demonstrated a stronger radical-scavenging activity than that of the positive control. Compounds **7** and **11** displayed some antioxidative effect toward radical-scavenging activity. Other compounds are inactive or weak at scavenging DPPH radicals at the tested conditions (Figure 5B).

### 2.4. Anti-Inflammatory Effects of Isolated Compounds

**Figure 6:**
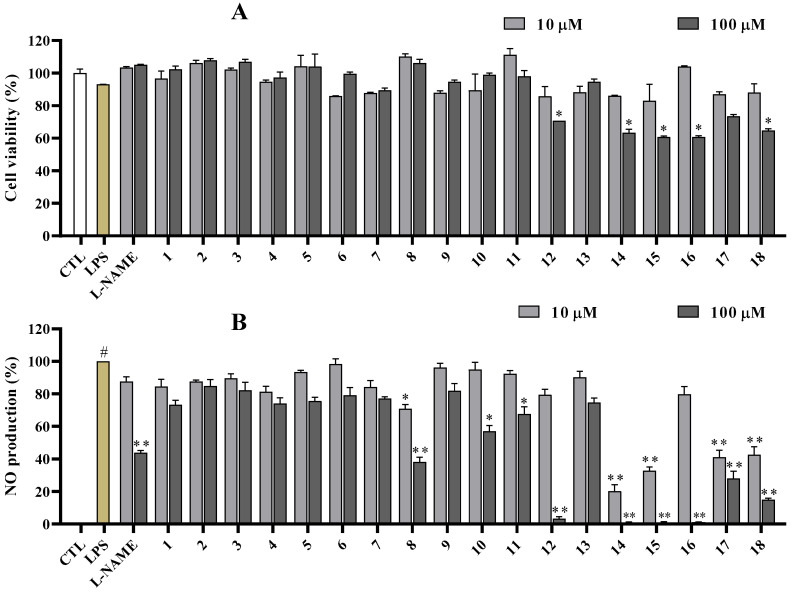
Anti-inflammatory effects of isolated compounds. RAW 264.7 cells were treated with samples for one hour before stimulating with LPS (1 µg/mL) for 16 h. (**A**) Cell viability was detected using MTT assay. (**B**) NO production was detected at 550 nm. Sample concentrations were tested at 10 and 100 μM. Each experiment was tested three times. The data are represented as mean ± SD. * *p* < 0.05, ** *p* < 0.01 vs. LPS treated group (yellow bar). ^#^ *p* < 0.01 vs. control (white bar).

The isolated compounds (**1**–**18**) regulated the expression levels of NO, IL-6, and TNF-α production in LPS-stimulated RAW264.7 cells (Figure 6). The control bar did not activate NO production in the absence of LPS addition. The LPS bar showed strong NO production after cells were activated by LPS. At 100 µM, all tested compounds showed NO production compared to the CTL bar, and their NO production was regulated by comparing it to that of LPS stimulation. Compounds (**8** and **12**–**18**) demonstrated a strong reduction in LPS-secreted NO production in RAW264.7 cells. Compounds **12** and **14**–**16** strongly inhibited NO production, with inhibition rates ranging from 99.04 ± 0.48% to 95.84 ± 2.38%. Compounds **8**, **17**, and **18** significantly reduced NO production, with inhibition rates of 61.86 ± 2.99%, 71.99 ± 0.04%, and 85.06 ± 0.92%, respectively, compared to the LPS bar without sample addition. Other compounds showed inactive or weak inhibition of LPS-induced NO production in RAW264.7 cells. However, almost all compounds did not affect cell viability, except compounds **14**–**16** and **18** at concentrations up to 100 µM. At 10 µM, compounds **14**, **15**, **17**, and **18** showed strong inhibition toward NO production, with inhibition rates of 79.74 ± 3.85%, 67.74 ± 4.75%, 58.98 ± 4.43%, and 57.38 ± 4.93%, respectively. Other compounds and the positive control exhibited weak or inactive activity at a concentration of 10 µM.

**Figure 7:**
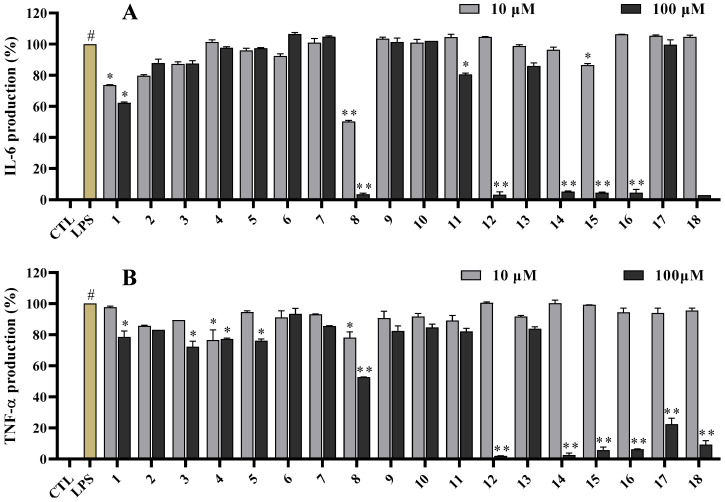
Inhibition of compounds on IL-6 production (**A**) and TNF-α production (**B**) induced by LPS-activated RAW264.7 cells. Sample concentrations were tested at 10 and 100 μM. Each experiment was tested three times. The data are represented as mean ± SD. * *p* < 0.05, ** *p* < 0.01 vs. LPS treated group (yellow bar). ^#^ *p* < 0.01 vs. control (white bar).

These isolated compounds also regulated IL-6 and TNF-α production secreted by LPS-stimulated RAW264.7 cells. Among them, compound **8** significantly inhibited IL-6 and TNF-α production compared to the LPS bar at both tested concentrations. At 100 µM, compounds **12**, **14**–**16**, and **18** showed significant reduction of IL-6 and TNF-α production compared to those of the LPS bar, with inhibition rates ranging from 97.82 ± 3.69% to 90.73 ± 2.41%. Compound **17** significantly inhibited TNF-α production, with an inhibition rate of 77.70 ± 3.08%, compared to the non-compound treatment of the LPS bar (Figure 7). The low variation between replicates supports the reliability of these findings.

### 2.5. Suppression of iNOS and COX-2 Enzymatic Proteins

**Figure 8:**
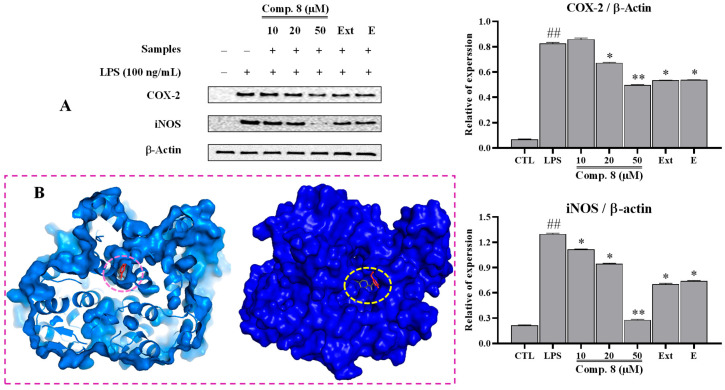
(**A**) Effects of compound **8**, extract (Ext), and EtOAc (E) fraction on COX-2 and iNOS expression. RAW 264.7 cells were incubated in the presence of samples for 1 h before incubating with LPS (1 µg/mL) for 16 h. The expression levels of iNOS, β-actin, and COX-2 were analyzed by Western blot analysis. Sample concentrations were tested at 10, 20, and 100 μM for compound **8** and at 50 μg/mL for Ext and E fraction. * *p* < 0.05, ** *p* < 0.01 vs. LPS-treated group. ^##^ *p* < 0.01 vs. control. (**B**) Compound **8** docked into the binding pocket of COX-2 and iNOS proteins.

The active compound (**8**), a potent anti-inflammatory, was further investigated in its action mode by regulating the expression levels of enzymatic proteins (iNOS and COX-2). In the Western blotting assay, both of the control bars did not show significant expression levels compared to the LPS treatment. For COX-2 expressions, the extract and EtOAc fractions reduced the COX-2 secretion in RAW264.7 cells compared to LPS bar stimulation. Compound **8** significantly reduced COX-2 exposure in a dose-dependent manner at concentrations of 10, 20, and 50 µM. For iNOS expression, by treating with the stimulator, the extract and EtOAc fraction also downregulated iNOS expression compared to LPS stimulation without sample addition. Compound **8** dose-dependently inhibited iNOS expression. Especially, compound **8** showed stronger inhibition toward the iNOS expression level than that of COX-2 at 50 µM. All the samples did not have any effect on the internal standard (β-actin) (Figure 8A). These findings revealed that hydroxytyrosol (**8**) might suppress inflammation by downstreaming the activity of enzymatic proteins (iNOS and COX-2) in the limiting steps during the production of inflammatory mediators.

### 2.6. Compound ***8*** Suppressed ERRK 1/2 MAPK Signaling

**Figure 9:**
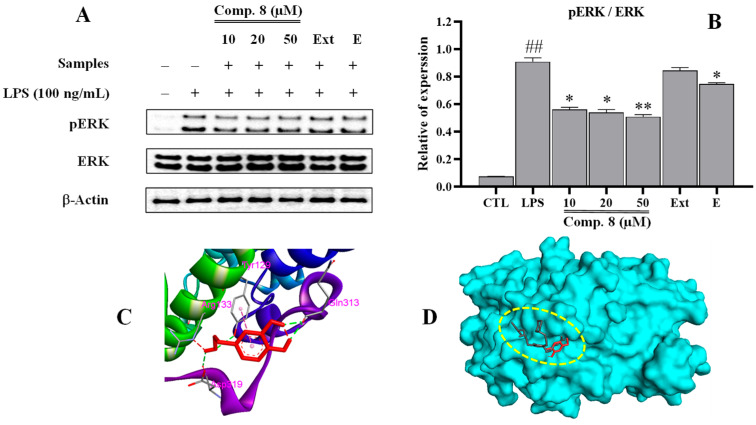
Effect of hydroxytyrosol (**8**) on pERK1/2 expression. RAW 264.7 cells were treated with samples at different concentrations for 1 h before stimulating with LPS (1 µg/mL) for 16 h. (**A**) The expression levels of ERK 1/2 and pERK1/2 were calculated by Western blot analysis. (**B**) Relative density was determined according to their detection compared to that of β-actin. Sample concentrations were tested at 10, 20, and 100 μM for compound **8** and at 50 μg/mL for Ext and E fraction. * *p* < 0.05, ** *p* < 0.01 vs. LPS-treated group. ^##^ *p* < 0.01 vs. control. (**C**) Interactions and (**D**) occupation of compound **8** with residues at the binding pose of ERK1/2 protein.

To further investigate the inhibitory effect, compound **8** was examined for activity in the ERK 1/2 MAPK signaling pathway. The control bar did not show the significance of pERK 1/2 expression. After treating the LPS stimulus, the phosphorylated ERK 1/2 expression was released compared to the control bar without LPS stimulation. Compound **8** inhibited the phosphorylation levels of pERK 1/2 stimulated by LPS. The E fraction and extract also reduced pERK 1/2 phosphorylation levels with significant inhibition compared to the LPS lane. The inhibitory effect of compound **8** is stronger than that of the E fraction and extract toward pERK 1/2 phosphorylation under the tested conditions (Figure 9A,B). Therefore, hydroxytyrosol may decrease NO production by inhibiting iNOS and COX-2 expression through the ERK 1/2 MAPK signaling pathway.

### 2.7. Molecular Docking Analysis

**Figure 10:**
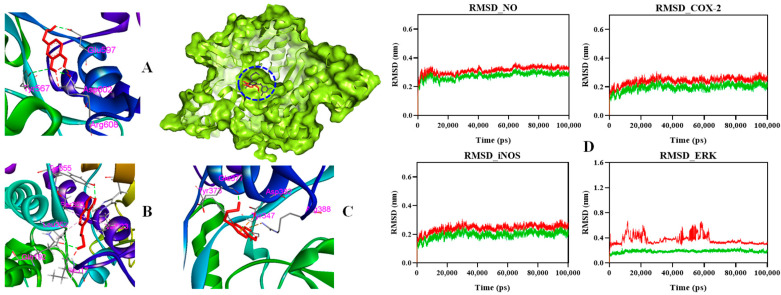
Visualization of 3D interactions of compound **8** and target proteins [nNOS (**A**), COX-2 (**B**), iNOS (**C**), green-dashed line represent hydrogen bond] and RMSD profiles (**D**) of the nNOS, COX-2, iNOS, and ERK protein–ligand complexes during a 100 ns molecular dynamics simulation (protein: green line; protein-ligand complex: red line).

An in silico assay was employed to predict the binding affinity of compound 8 toward target proteins [nNOS (pdb ID: 6AV2), COX-2 (pdb ID: 5IKQ), ERK (pdb ID: 6NBS), and iNOS (pdb ID: 3E7G)]. At first, all the co-crystal structures of the above-mentioned proteins were re-docked to validate the docking protocol, showing the root mean square deviation (RMSD)to be less than 2.0 Å. Compound **8** was docked into the binding pocket of the target proteins (Figure 8B, Figure 9D and Figure 10A). This observation revealed the accuracy of the docking method. Subsequently, compound **8** was docked into the nNOS protein and showed a docking score of −5.58 kcal/mol. Compound **8** may establish hydrogen bond interactions with TYR567, GLU597, and ASP602 [29], key residues of the binding pocket of nNOS protein (Figure 9A). When compound **8** docked into the COX-2 protein, it showed a binding energy of −7.38 kcal/mol through interacting with the key amino acid TYR355 and with GLN192, by forming hydrogen bonds. When compound **8** docked into the iNOS protein, it showed a binding score of −5.35 kcal/mol, interacted with the protein by forming hydrogen bonds with key amino acids (Figure 8B), TYR347, TYR373, and GLY377 [30]. Similarly, compound **8** also displayed a docked score of −4.93 kcal/mol to the ERK protein and showed interactions with residues, including TYR129, GLN313, and ASP319 (Figure 9B), in the binding pose of the established complex. The molecular docking approach provides evidence for interactions between compound **8** and proteins that target inflammation through both binding affinity and interactions with key residues of the binding pose within ligand–protein complexes.

### 2.8. Molecular Dynamics Simulation Analysis

To investigate the complex stability and interaction profiles of compound **8** inside the active sites of the target proteins, nNOS, iNOS, COX-2, and ERK, compound **8** simultaneously interacted with each protein during simulation time. The structural parameters, such as RMSD, RMSF, SASA, Rg, and H-bond, were evaluated as a function of time. As shown in the figure, all complexes achieved equilibration within the first 10–20 ns, and all complexes showed conformational stability over a 100 ns time frame, suggesting that compound **8** has favorable and stable interactions with all target proteins. Briefly, compound **8** and the COX-2 complex exhibited the most stable profile, with the RMSD values remaining consistently around 0.2–0.3 Å. The complexes of compound **8** docked with the nNOS and iNOS protein revealed modest deviation around 0.25–0.4 Å, indicating stable ligand accommodation in the complexes. Additionally, ERK displayed higher early fluctuations (up to 0.6 nm) for the ligand-bound form, yet the protein backbone RMSD remained low (~0.2 nm), indicating minor ligand movement without disrupting protein structure (Figure 10D).

**Figure 11:**
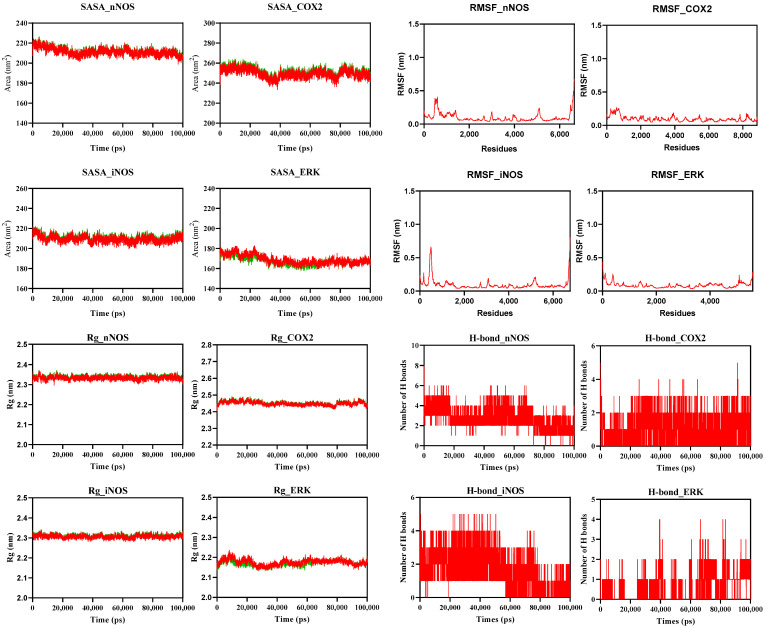
SASA, RMSF, Rg, and H-bond profiles of the nNOS, COX-2, iNOS, and ERK protein–ligand complexes during a 100 ns molecular dynamics simulation (protein: green line; protein-ligand complex: red line).

The solvent-accessible surface area (SASA) analysis offers insights into the dynamic behavior and structural compaction of the protein complexes during molecular dynamics simulations. As illustrated in Figure 11, the nNOS and iNOS systems exhibited relatively stable SASA values (200–230 nm^2^). The COX-2 complex exhibited some decrease in SASA (260 to 240 nm^2^) during the initial half of the simulation, indicating a possible conformational rearrangement or folding event, reducing the protein’s surface exposure (Figure 11). The ERK complex experienced an initial drop in SASA, stabilizing afterward around 190 nm^2^, which may reflect an equilibration phase, leading to a more compact structure. These trends, in alignment with the radius of gyration (Rg) analysis, support the conclusion that the simulated proteins generally retained their structural integrity, with COX-2 and ERK undergoing slight but stable compaction throughout the trajectory.

The flexibility of each residue during the simulation is revealed by the RMSF analysis. Most of the residues in all four protein–ligand complexes have modest fluctuation values (usually less than 0.3 nm), suggesting a generally stable conformation (Figure 11). The RMSF profiles of the nNOS and iNOS complexes show localized peaks, which may indicate flexible loop segments or terminal residues that are not directly involved in ligand binding. In contrast, COX-2 and ERK exhibit continuously low RMSF values across almost the whole sequence. These results align with a previously observed low radius of the below data of gyration (Rg) and stable SASA values, reinforcing the idea that these complexes maintain compact and structurally coherent conformations.

Protein systems’ structural stability and compactness can be inferred from the radius of gyration (Rg) in molecular dynamics simulations. Figure 11 showed that, throughout the 100 ns simulation, the Rg profiles of the four target proteins, nNOS, COX-2, iNOS, and ERK, remained rather consistent. nNOS and iNOS complexes demonstrated a stable compactness, with Rg values of 2.30–2.35 nm, indicating high structural integrity and minimal conformational change. Overall, our results suggest that the simulated protein systems retained their structural compactness, supporting the correctness of the molecular dynamics data and the potential stability of ligand–target interactions.

An essential component of complex stability is hydrogen bonding. Particularly in the initial 60–70 ns of the simulations, the nNOS and iNOS complexes displayed a significant number of hydrogen bonds, with nNOS continuously retaining between three and six hydrogen bonds (Figure 11). This suggests that the ligands and certain targets have a comparatively robust interaction. The iNOS complex also showed a strong hydrogen bonding network. However, a decrease was observed toward the end, which might have been caused by small conformational changes. The COX-2 complex maintains a fluctuation of hydrogen bonds around 1 and 3, which suggests a more flexible interaction. Low hydrogen bonding was seen in the ERK complex, suggesting a dynamic complex. In these findings, particularly in the initial 60–70 ns of the simulations, the nNOS and iNOS complexes displayed a significant number of hydrogen bonds, with nNOS continuously retaining between three and six hydrogen bonds (Figure 11). This suggests that the ligands and certain targets have a comparatively robust and long-lasting interaction.

## 3. Discussion

A phytochemical investigation led to the isolation and identification of 18 compounds, including one new compound, two lignans, seven phenolics, one secoiridoid, and seven triterpenoid derivatives from the ethanolic extract of *O. fragrans* leaves. The structure of fragrans D1 was successfully determined using modern techniques, such as spectroscopic, CD, acid hydrolysis, and HPLC analysis. Our study first reported the isolation of syringin and 8-epikingiside from the leaves of this plant. *O. fragrans* has potent anti-inflammatory activity by modulating pro-inflammatory cytokines through different signaling pathways by suppressing COX-2 and iNOS and NF-κB activation [22,31]. These properties suggested *O. fragrans* as a promising natural source for managing oxidative and inflammatory conditions. The ethnopharmacological use of *O. fragrans* suggests that a variety of bioactive compounds within the plant may contribute to its perceived medicinal efficacy [32]. According to previous research, chemical constituents identified from *O. fragrans* supported its traditional anti-inflammatory usage [6] and synthesized the available in vitro, in vivo, and clinical evidence [8], indicating that it may be a source of materials for inflammatory disease approaches. Indeed, the extract and fractions of this plant potentially showed anti-inflammatory and antioxidative effects. Therefore, GNPS-FBMN guidance is used to clarify the anti-inflammatory potential of this herbal medicine by identifying the active components responsible for identifying compound **8** with a high anti-inflammatory effect.

Subsequently, these isolated compounds were assessed for their effect on reducing NO production induced by LPS-activated RAW264.7 cells. According to the anti-inflammatory effect of these compounds, a structure–activity relationship was concluded. Compounds **2**, **3**, and **8** shared the 3,4-dihydroxy-phenyl derivatives. However, compound **8** showed much stronger inhibition in LPS-induced NO production than those of **2** and **3**. This observation suggests that the hydroxyethyl functional group is important to promote anti-inflammatory effects under the experimental conditions. Compounds (**9**–**11**) are furan lignan compounds. However, compound **11** showed a little stronger activity than that of **9** and **10**, revealing that the glycosylation of this compound group is not favored for inhibiting NO production induced by LPS-stimulating RAW264.7 cells. Our study is the first report of compounds **4**, **5**, **7**, and **9** having an inhibitory effect on NO production in LPS-stimulated RAW264.7 cells.

Nitric oxide (NO) is a reactive gaseous signaling molecule produced by various isoforms of nitric oxide synthase (NOS), with iNOS being primarily responsible for the high-output NO production seen during inflammation. The excessive production of NO has been implicated in oxidative stress, tissue damage, and the perpetuation of chronic inflammation. iNOS is typically undetectable under normal conditions but is robustly induced in macrophages, epithelial cells, and other immune cells in response to inflammatory stimuli such as lipopolysaccharides (LPS), tumor necrosis factor-α (TNF-α), and interferon-γ (IFN-γ). Similarly, COX-2, an inducible isoform of the cyclooxygenase enzyme family, is upregulated in response to inflammatory cytokines and contributes to the synthesis of prostaglandins, particularly prostaglandin E_2_ (PGE_2_), which enhances vascular permeability, pain, and leukocyte recruitment. COX-2 expression is a hallmark of inflamed tissues and has become a well-recognized target for anti-inflammatory drugs, including selective COX-2 inhibitors (coxibs). Given their central roles in the inflammatory response, iNOS and COX-2 have emerged as critical therapeutic targets. Modulation of their expression or activity has been shown to attenuate inflammation and tissue injury in a variety of experimental models [33]. The active compound exhibited an inhibitory effect on iNOS and COX-2 expression. This observation suggested that compound **8** may suppress NO production by the downstream enzymatic activity during the step to produce inflammatory mediators.

Inflammatory stimuli such as lipopolysaccharides (LPS) and cytokines can activate the ERK 1/2 MAPK cascade, which in turn promotes the transcriptional upregulation of iNOS, leading to increased NO production. On the other hand, excessive NO, particularly when derived from iNOS, can modulate MAPK activity through S-nitrosylation of upstream kinases, cGMP-dependent pathways, or oxidative stress mechanisms involving peroxynitrite. These interactions can either amplify or attenuate ERK 1/2 signaling, depending on the cellular context and the redox balance [34,35]. Furthermore, this active compound was also found to decrease the phosphorylation level of ERK 1/2 MAPK. On the other hand, the activation of pERK 1/2 MAPK leads to increased expression of COX-2 and iNOS enzymes, thereby increasing inflammatory mediators, which contribute to fever, pain, inflammation, and tissue damage [36]. Compound **8** significantly inhibits LPS-induced ERK 1/2 phosphorylation, a key step in the MAPK inflammatory pathway.

In silico approaches provided the evidence supporting the activities of compound **8** to COX-2, iNOS, and ERK 1/2 MAPK by its good binding affinity to each protein and interacting with key residues at the active sites of the target protein, respectively. The molecular dynamics simulations reveal that all four ligands form stable complexes with their respective target proteins. COX-2 and ERK demonstrated the most compact and stable dynamics, while nNOS and iNOS maintained consistent binding interactions. The combination of RMSD, RMSF, Rg, SASA, and hydrogen bond data provides robust evidence for ligand compatibility and binding stability, supporting further investigation into their therapeutic potential. Collectively, experimental and in silico approaches suggested that hydroxytyrosol exhibits potential for use as a pharmacological lead or anti-inflammatory nutraceutical targeting ERK MAPK signaling. Further studies should be conducted on higher experimental models and compound metabolism-altering efficacy to validate the potential found in this study. Our findings confirm prior research that has shown that hydroxytyrosol is potent in a wide range of applications for the prevention of inflammation, oxidation, and apoptosis [35]. Future research should also investigate the optimal dosages and delivery methods to maximize its bioavailability and effectiveness in clinical settings.

Furthermore, these compounds also displayed an antioxidative effect to DPPH and ABTS radical-scavenging activity. Based on their antioxidant activity, a structure–activity relationship proposed that compounds **6**–**8** may be represented for their own fraction, and the extract potentially scavenged both radicals. A structure–activity relationship revealed that compounds **2** and **3** are phenols with a difference in the CH_3_ functional group. Compound **2** showed a stronger radical-scavenging activity than that of **3**. Thus, the OH functional group is more important for donating hydrogen atoms or transferring electrons than the CH_3_ functional group. Compounds **9**–**11** belong to furan lignan derivatives. However, compound **9** showed less radical-scavenging activity than those of **10** and **11**. This observation suggested that the 7*R* conformation of the furan moiety is crucial to promote radical-scavenging activity. This is the first report for the radical scavenging activities of **4**, **5**, **7**, **9**, **17**, and **18**.

## 4. Materials and Methods

### 4.1. General Procedures

The NMR spectra of isolates **1**–**18** were recorded on a JEOL JNM-ECZ 600 MHz spectrometer (JEOL, Tokyo, Japan), and chemical shifts were expressed as *δ* values (ppm) with TMS as the internal standard (measured in methanol-*d*_4_). CD spectra were obtained using a JASCO J-1500 spectrophotometer (JASCO Corporation, Tokyo, Japan). The mass confirmation was performed on a Thermo Scientific Vanquish UHPLC connected with an Orbitrap 120 mass spectrometer system (Thermo Fisher Scientific, Sunnyvale, CA, USA).

### 4.2. Extraction and Separation of Compounds

The *O. fragrans* leaves were collected from Sunchon National University Forests (Suncheon, Republic of Korea) on a sunny day on 10 September 2023. Professor Mina Lee identified the botanical specimen of this plant. A voucher specimen (SCNUP21) was stored at the laboratory of Pharmacognosy, College of Pharmacy, Sunchon National University (Suncheon, Republic of Korea). The dried leaves (3.93 kg) of *O. fragrans* were ground into a powder before being extracted with methanol at room temperature for 90 min. The solution was concentrated under a vacuum to yield an extract (700 g). This extract was partitioned step by step using an increasing polarity of the solvents Hex (43.19 g), MC (100.93 g), E (70.26 g), Bu (560.61 g), and W (146.09 g) fractions, respectively. The E fraction was inserted into open column chromatography using ODS silica gel (Fuji Silisa Chemical Ltd., Kasugai, Aichi, Japan), eluting methanol in distilled water by a gradient solvent system from 20 to 100% to obtain 50 fractions (E1–E50). Subfraction E3 was subjected to a prep HPLC using a YMC-Triart C_18_ column (10 × 250 mm, 5 µm) and flow rate 3.0 mL/min, and detected at wavelength 255 nm using a mobile phase of water (buffering 0.1% formic acid, A) and methanol (B) as a gradient solvent system from 12% B to 100% B during 90 min to obtain **1** (*t*_R_ 38 min), **2** (*t*_R_ 48 min), **3** (*t*_R_ 55 min), **4** (*t*_R_ 35 min), **6** (*t*_R_ 69 min), **7** (*t*_R_ 88 min), and **8** (*t*_R_ 21 min), in which each peak was determined based on its UV adsorption and relative retention time. The separation of the E3 subfraction was conducted by repeating the procedures six times. Similarly, compounds **5** (*t*_R_ 49.1 min), **9** (*t*_R_ 38.9 min), and **10** (*t*_R_ 43 min) were isolated from the E11 sub-fraction three times by repeating the experiments using a prep-HPLC using a Triart C_18_ (10 × 250 mm, 5 µm), with a flow rate of 3.0 mL/min, detected at wavelength 265 nm, eluting with a mobile phase consisting of water (buffered with 0.1% formic acid, A) and methanol (B) using an isocratic elution of 42%, B from 0 to 70 min. Subfraction E35 was purified five times by preparative HPLC on a YMC-Triart C_18_ column (Triart C_18_, 10 × 250 mm, 5 μm, YMC, Tokyo, Japan) using a gradient mobile phase of 0.1% formic acid in water (A) and methanol (B) (12–100% B over 90 min) at 3.0 mL/min, with detection at 255 nm to obtain compound **18** (*t*_R_ 54 min). In addition, the MC fraction was injected into an MPLC [consisting of methanol in water (flow rate of 15 mL/min; buffered with 0.1% formic acid) from 30% to 100% for 90 min; UV at 210 and 254 nm; Column C_18_ 120 g (Biotage, Uppsala, Sweden)] to obtain fifteen sub-fractions (MC1–MC15) by repeating the procedure 20 times. Subfraction MC13 (1.18 g) was separated ten times by using HPLC using a flow rate 3.0 mL/min, column (Triart C_18_, 10 × 250 mm, 5 μm, YMC, Tokyo, Japan), UV at 254 nm and 310 nm, eluting with a gradient mobile phase of water (buffered with 0.1% formic acid, (A) and methanol (B) as follows 0 min (80% B)–80 min (85% B)–90 min (100% B)) to obtain compounds **12** (*t*_R_ 82 min), **13** (*t*_R_ 43 min), **14** (*t*_R_ 74 min), **15** (*t*_R_ 84 min), **16** (*t*_R_ 47 min), and **17** (*t*_R_ 63 min), and further isolated under the same conditions by an isocratic elution of 80% B for 30 min, for compound **11** (*t*_R_ 12 min).

#### Chemical Information of Compounds

Syringin (**1**): While amorphous powder; MF: C_17_H_24_O_9_; HR-ESI-MS *m*/*z* 390.1740 [M + NH_4_]^+^. Smile: COC1=CC(=CC(=C1O[C@H]2[C@@H]([C@H]([C@@H]([C@H](O2)CO)O)O)O)OC)/C=C/CO.

Caffeic acid (**2**): While amorphous powder; MF: C_9_H_8_O_4_; HR-ESI-MS *m*/*z* 179.0343 [M − H]^−^. Smile: C1=CC(=C(C=C1/C=C/C(=O)O)O)O.

Ferulic acid (**3**): While amorphous powder; MF: C_10_H_10_O_4_; HR-ESI-MS *m*/*z* 193.0502 [M − H]^−^. Smile: COC1=C(C=CC(=C1)/C=C/C(=O)O)O.

8-Epikingiside (**4**): While amorphous powder; MF: C_17_H_24_O_11_; HR-ESI-MS *m*/*z* 405.1368 [M + H]^+^. Smile: C[C@@H]1[C@@H]2[C@H](CC(=O)O1)C(=CO[C@H]2O[C@H]3[C@@H]([C@H]([C@@H]([C@H](O3)CO)O)O)O)C(=O)OC.

10-Acetoxyligustroside (**5**): While amorphous powder; MF: C_27_H_34_O_14_; HR-ESI-MS *m*/*z* 581.1851 [M − H]^−^. Smile: CC(=O)OC/C=C/1\[C@@H](C(=CO[C@H]1O[C@H]2[C@@H]([C@H]([C@@H]([C@H](O2)CO)O)O)O)C(=O)OC)CC(=O)OCCC3=CC=C(C=C3)O.

Verbacoside (**6**): While amorphous powder; MF: C_29_H_36_O_15_; HR-ESI-MS *m/z* 623.1957 [M − H]^−^. Smile: C[C@H]1[C@@H]([C@H]([C@H]([C@@H](O1)O[C@@H]2[C@H]([C@@H](O[C@@H]([C@H]2OC(=O)/C=C/C3=CC(=C(C=C3)O)O)CO)OCCC4=CC(=C(C=C4)O)O)O)O)O)O.

10-Acetoxyoleuropein (**7**): While amorphous powder; MF: C_27_H_34_O_15_; HR-ESI-MS *m*/*z* 597.1805 [M − H]^−^. Smile: CC(=O)OC/C=C/1\C(C(=COC1OC2C(C(C(C(O2)CO)O)O)O)C(=O)OC)CC(=O)OCCC3=CC(=C(C=C3)O)O.

Hydroxytyrosol (**8**): While amorphous powder; MF: C_8_H_10_O_3_; HR-ESI-MS *m*/*z* 153.0550 [M − H]^−^. Smile: C1=CC(=C(C=C1CCO)O)O.

New compound (**9**): While amorphous powder; MF: C_27_H_34_O_11_; HR-ESI-MS *m*/*z* 552.2410 [M + NH_4_]^+^. Smile: O[C@H]1[C@H](OC2=C(OC)C=C([C@H]3[C@]([C@@]4([H])CO3)([H])CO[C@@H]4C5=CC(OC)=C(OC)C=C5)C=C2)O[C@H](CO)[C@@H](O)[C@@H]1O.

Phillyrin (**10**): While amorphous powder; MF: C_27_H_34_O_11_; HR-ESI-MS *m*/*z* 552.2411 [M + NH_4_]^+^. Smile: COC1=C(C=C(C=C1)[C@H]2[C@H]3CO[C@@H]([C@H]3CO2)C4=CC(=C(C=C4)O[C@H]5[C@@H]([C@H]([C@@H]([C@H](O5)CO)O)O)O)OC)OC.

(+)-phillygenin (**11**): While amorphous powder; MF: C_21_H_24_O_6_; HR-ESI-MS *m*/*z* 390.1610 [M + NH_4_]^+^. Smile: COC1=C(C=C(C=C1)[C@H]2[C@H]3CO[C@@H]([C@H]3CO2)C4=CC(=C(C=C4)O)OC)OC.

Oleanonic acid (**12**): While amorphous powder; MF: C_30_H_46_O_3_; HR-ESI-MS *m*/*z* 472.3477 [M + NH_4_]^+^. Smile: C[C@]12CCC(=O)C([C@@H]1CC[C@@]3([C@@H]2CC=C4[C@]3(CC[C@@]5([C@H]4CC(CC5)(C)C)C(=O)O)C)C)(C)C.

Maslinic acid (**13**): While amorphous powder; MF: C_30_H_48_O_4_; HR-ESI-MS *m*/*z* 455.3496 [M − NH_4_]^−^. Smile: C[C@@]12CC[C@@H]3[C@@]([C@H]1CC=C4[C@]2(CC[C@@]5([C@H]4CC(CC5)(C)C)C(=O)O)C)(C[C@H]([C@@H](C3(C)C)O)O)C.

3-*O-cis*-coumaroylmaslinic acid (**14**): While amorphous powder; MF: C_39_H_54_O_6_; HR-ESI-MS *m*/*z* 619.3961 [M + H]^+^. Smile: CC1(CCC2(CCC3(C(=CCC4C3(CCC5C4(CC(C(C5(C)C)OC(=O)/C=C\C6=CC=C(C=C6)O)O)C)C)C2C1)C)C(=O)O)C.

Ursolic acid (**15**): While amorphous powder; MF: C_30_H_48_O_3_; HR-ESI-MS 457.3288 [M + H]^+^. Smile: C[C@@H]1CC[C@@]2(CC[C@@]3(C(=CC[C@H]4[C@]3(CC[C@@H]5[C@@]4(CC[C@@H](C5(C)C)O)C)C)[C@@H]2[C@H]1C)C)C(=O)O.

Corosolic acid (**16**): While amorphous powder; MF: C_30_H_48_O_4_; HR-ESI-MS *m*/*z* 471.3461 [M − H]^−^. Smile: C[C@@H]1CC[C@@]2(CC[C@@]3(C(=CC[C@H]4[C@]3(CC[C@@H]5[C@@]4(C[C@H]([C@@H](C5(C)C)O)O)C)C)[C@@H]2[C@H]1C)C)C(=O)O.

3*β*-*cis*-*p*-coumaroyloxy-2*α*-hydroxyl-urs-12-en-28-oic acid (**17**): While amorphous powder; MF: C_39_H_54_O_6_; HR-ESI-MS *m*/*z* 619.3961 [M + H]^+^. Smile:C[C@@H]1CC[C@@]2(CC[C@@]3(C(=CC[C@H]4[C@]3(CC[C@@H]5[C@@]4(C[C@H]([C@@H](C5(C)C)O)O)C)C)[C@@H]2[C@H]1C)C)C(=O)O.

3*β*-*trans*-*p*-coumaroyloxy-2*α*-hydroxyl-urs-12-en-28-oic acid (**18**): While amorphous powder; MF: C_39_H_54_O_6_; HR-ESI-MS *m*/*z* 619.3965 [M + H]^+^. Smile:C[C@]12[C@]3(C)CC[C@@]4([H])C(C)(C)[C@@H](OC(/C=C/C5=CC=C(O)C=C5)=O)[C@H](O)C[C@]4(C)C3CC=C1[C@@]6([H])[C@@](CC[C@@H](C)[C@@H]6C)(C(O)=O)CC2.

### 4.3. Biological Assays

#### 4.3.1. Antioxidative Assays

DPPH and ABTS radical-scavenging assays were used to evaluate the antioxidant effect of the extract, fractions, and compounds. In the DPPH assay, the reaction mixture was measured for the absorbance using a micro reader (Epoch, Biotek Instruments, Inc., Winooski, VT, USA) at 517 nm after incubating at room temperature for 30 min in the dark. In the ABTS assay, the reaction mixture was measured for absorbance at 734 nm after reacting in the dark for 20 min. Negative controls (CTL) were prepared by replacing the sample with the same volume of solvent. The positive control (AA) for both assays was ascorbic acid. Samples were prepared at concentrations of 10 and 100 μg/mL for the extract and fractions, and at concentrations of 10 and 100 μM for compounds.

#### 4.3.2. Cell Culture and Viability

The Korean Cell Lines Bank (Seoul, Republic of Korea) supplied RAW 264.7 cells. Cells were grown at 37 °C in DMEM supplemented with 10% heat-inactivated FBS, streptomycin sulfate (100 μg/mL), and penicillin (100 IU/mL) in a humidified atmosphere of 5% CO_2_. After pre-incubation of the RAW264.7 cells for 1 h, each extract, fraction, and compound was added to the working solution. RAW 264.7 cells’ viability was assessed for 24 h by using an MTT (Dojindo, Tokyo, Japan) assay at 540 nm. Survival cells were determined by using the following formula: viable cell number (%) = OD_540_ (treated cell culture) × 100 /OD_540_ (vehicle control) [37].

#### 4.3.3. NO Production Determination

The generation of NO production was assessed by determining the nitrite amount generated from supernatants of the cell culture. Here, 100 μL of culture supernatant was transferred to a new 96-well plate and mixed with an equal volume of Griess reagent, prepared by combining equal volumes of 0.1% (*w*/*v*) N-(1-naphtyl) ethylenediamine (Griess reagent A) and 1% (*w*/*v*) sulfanilamide in 5% (*v*/*v*) phosphoric acid (Griess reagent B). The mixture was stirred for 15 min at room temperature. NO production was determined by their absorbance at 550 nm. The positive control was L-NAME (Sigma-Aldrich, Co., St. Louis, MO, USA).

#### 4.3.4. Measurement of IL-6 and TNF-α Production

The IL-6 and TNF-α production induced by LPS-activated RAW264.7 cells was determined by using the ELISA kit (BD OptEIATM, San Jose, CA, USA) according to the manufacturer’s instructions by following our previous method [38].

#### 4.3.5. Western Blotting

RAW264.7 cells were pretreated for 2 h before being stimulated with LPS (100 ng/mL) for a specified duration. Then, cells were lysed with PRO-PREPTM (Intron Biotechnology, Seoul, Republic of Korea) supplemented using the Pierce™ Bradford Protein Assay Kit (Pierce™ Bradford Protein Assay Kit, Waltham, MA, USA). BD Bioscience (San Jose, CA, USA) provided β-actin (AB_2289199), iNOS (AB_397808), and COX-2 (AB_397603). Cell Signaling Technology provided anti-p44/42 MAPK (ERK 1/2) (1:1000; cat. No. 4695) and anti-phospho p44/42 MAPK (ERK 1/2) (1:1000; cat. No. 9101). The blots were placed on PVDF membranes, blocked for two hours with 5% skim milk, then incubated for eighteen hours at 4 °C with the primary antibodies diluted 1:1000 in 2.5% skim milk. They were then washed three times with Tween20/Tris-buffered saline (T/TBS) and incubated for two hours at room temperature with an HRP-conjugated secondary antibody diluted 1:2000 in 5% skim milk and then again with T/TBS. Using the Super Signal ™ West Femto Maximum Sensitivity Substrate (Thermo Fisher Scientific, Waltham, MA, USA), the expression of the desired protein was found. The band intensity was measured by MicroChemi 4.2 with 10 s of exposure time.

### 4.4. Molecular Docking

The binding affinity of compound **8** to the target proteins [nNOS (pdb ID: 6AV2), COX-2 (pdb ID: 5IKQ), ERK (pdb ID: 6NBS) [38], and iNOS (pdb ID: 3E7G), all accessed on June 18, 2025] were predicted using in silico experiments. MGL Tools 1.5.6 (The Scripps Research Institute, La Jolla, CA, USA) was used to build the structures of the proteins and ligands. By removing water molecules, adding polar hydrogen atoms, and assigning Kollman charges, the receptors were prepared. The structure of compound **8** was retrieved in sdf format from PubChem (https://pubchem.ncbi.nlm.nih.gov; accessed on 23 June 2025) and converted to pdbqt format using the Open Babel tool. Before utilizing the AutoGrid tool, ligands were given Gasteiger charges, and torsions were adjusted. The grid box coordinates were established by using PyMOL. These dimension coordinates showed the centroid of the native ligand in the crystal structures or the important amino acids in the protein-binding pocket. We used AutoDock 4.2 to conduct protein–ligand docking calculations. The scoring function used van der Waals contacts, electrostatics, hydrogen bonding, desolvation effects, and torsional entropy contributions to make very accurate predictions about how the ligands would bind and how strong their bonds would be. Discovery Studio 2021 (BIOVIA, San Diego, CA, USA) and PyMOL (Version 4.6, Schrödinger, New York, NY, USA) were used to visualize the residues and ligand interactions.

### 4.5. Molecular Dynamics Simulation

Molecular dynamics (MD) simulations were performed using GROMACS 2023.3 to determine whether the docked complex is stable over time in a biologically simulated environment. The TIP3P water model and the AmberGS force field were chosen during topology preparation. Charges and hydrogen were added to the ligand and protein structures. The system was solved in a cubic simulation box using the SPC/E water model, with a buffer of 1 nm separating the protein from the box boundary. Energy minimization was conducted using the steepest descent algorithm for 100,000 steps. The system was then brought into balance under NVT (number of particles, volume, and temperature) conditions at 300 K. A V-rescale thermostat with a time constant of 0.1 ps was used to keep the temperature stable. The Berendsen barostat was used to conduct an NPT (constant number of particles, pressure, and temperature) equilibration to set the system pressure to 1 bar. The Parrinello–Rahman barostat was used to run production MD simulations under NPT conditions after the solute’s position was no longer restricted. As part of the trajectory analysis, we figured out the complex backbone RMSD, RMSF, SASA, H-bond, and Rg values [39].

### 4.6. Statistical Analysis

Data was expressed as the mean ± standard error based on three independent experiments (*n* = 3). We used one-way analysis of variance (ANOVA) and Dunnett’s post hoc test to compare the treatment groups with the control group. All analyses were conducted using GraphPad Prism software version 8.0 (GraphPad Software, Inc., San Diego, CA, USA). Statistical significance was defined as * *p* < 0.05, ** *p* < 0.01, and ^#^ *p* < 0.01, compared to the control group.

## 5. Conclusions

In summary, this study investigates the anti-inflammatory and antioxidative effects of *O. fragrans* by identifying the active constituents guided by GNPS-FBMN. A new compound, fragrans D1, and 17 known compounds were isolated and identified from the ethanolic extract of *O. fragrans*. These compounds exhibited antioxidative capacity toward DPPH and ATBS radicals. The isolates suppressed the NO production secreted by LPS-activated RAW264.7 cells in vitro studies. Our findings suggested that hydroxytyrosol has potent anti-inflammatory effects by the inactivation of ERK 1/2 MAPK signaling. Molecular docking validated the bioactivities of the active compounds. Molecular dynamics simulation confirmed the stability and compactness of the hydroxytyrosol–protein complexes targeting inflammation. Our findings reveal that active compounds may be promising candidates for the therapeutic efficiency of the anti-inflammatory properties of *O. fragrans* in traditional medicine. Hydroxytyrosol may be considered for developing functional products to manage the progression of inflammatory conditions in the future.

## Figures and Tables

**Table 1:** NMR spectroscopic data of compounds **1**, **4**, and **9**, measured in CD_3_OD.

	1		4		9	
Position	*δ*_H_ (mult., *J* = Hz)	*δ*_C_ (150 MHz)	*δ*_H_ (mult., *J* = Hz)	*δ*_C_ (150 MHz)	*δ*_H_ (mult., *J* = Hz)	*δ*_C_ (150 MHz)
1	-	134.5	5.50 (d, 7.7)	96.3	-	137.6
2	6.75 (s)	154.4	-	-	7.03 (d, 2.0)	111.6
3	-	105.5	7.58 (d, 1.2)	154.4	-	151.1
4	-	135.9	-	109.6	-	147.6
5	-	105.5	3.08 (dddd, 11.7, 7.2, 4.7, 1.3)	28.2	7.15 (d, 8.3)	118.6
6	6.75 (s)	154.4	2.51 (dd, 16.5, 11.4) 2.86 (ddd, 16.5, 4.7)	34.6	6.93 (dd, 8.3, 2.0)	119.3
7	6.55 (d, 15.8)	131.3	-	174.7	4.48 (d, 6.8)	89.1
8	6.33 (dt, 5.6, 15.8)	130.1	4.49 (m)	74.7	2.94 (m)	55.8
9	4.22 (dd, 1.7, 5.6)	63.0	2.14 (q, 7.0)	41.9	4.15 (dd, 9.3, 1.5) 3.88 (m)	72.1
10	-	-	1.51 (d, 6.4)	21.7	-	-
11	-	-	-	168.3	-	-
1′	4.87 (d, 7.7)	103.4	4.70 (d, 7.9)	100.7	-	132.8
2′	3.21 (m)	75.8	3.21 (dd, 6.8, 1.9)	75.8	7.01 (d, 1.8)	110.9
3′	3.15 (m)	77.9	3.33 (m)	78.6		150.4
4′	3.18 (m)	71.4	3.24 (m)	71.7		149.6
5′	3.21 (m)	77.9		77.9	6.94 (m) *	112.9
6′	3.78 (dd, 2.4, 11.9) 3.66 (dd, 5.3, 12.0)	62.6	3.92 (dd, 11.9, 2.2) 3.63 (dd, 11.9, 6.8)	62.9	6.94 (m) *	118.1
7′	-	-	-	-	4.89 (d, 5.9)	83.4
8′	-	-	-	-	3.41 (m)	51.3
9′	-	-	-	-	3.80 (m) 3.28 (dd, 9.3, 7.9)	70.7
1′′	-	-	-	-	4.88 (d, 7.4)	102.9
2′′	-	-	-	-	3.45–3.53 (m)	74.9
3′′	-	-	-	-	3.45–3.53 (m)	78.2
4′′	-	-	-	-	2.94 (m)	71.4
5′′	-	-	-	-	3.45–3.53 (m)	77.9
6′′	-	-	-	-	3.87 (m) 3.69 (m)	62.5
2-OCH_3_	3.86 (s)	57.0	-	-	-	-
3-OCH_3_	-	-	-	-	3.87 (s)	56.8
3′-OCH_3_	-	-	-	-	3.85 (s)	56.6
4′-OCH_3_	-	-	-	-	3.83 (s)	56.5
6-OCH_3_	3.86 (s)	57.0	-	-	-	-
11-OCH_3_	-	-	-	51.8	-	-

* Overlap signal.

## Data Availability

The original contributions presented in the study are included in the article/Supplementary Materials; further inquiries can be directed to the corresponding author.

## Supplementary Materials

The following supporting information can be downloaded at https://www.mdpi.com/article/10.3390/ijms26178421/s1.
