# Supplementary material for: Chemical Constituents from Osmanthus fragrans var. aurantiacus Makino with Their In Vitro and In Silico Studies Target Anti-Inflammation by Suppressing ERK 1/2 MAPK Signaling
Source: Int J Mol Sci. 2025 Aug 29;26(17):8421. doi: 10.3390/ijms26178421 (PMC12428955; doi:10.3390/ijms26178421)
Supplement: Supplementary file 1 [file ijms-26-08421-s001.zip › ijms-3755623-supplementary.pdf]

Chemical constituents from *Osmanthus fragrans* var. *Aurantiacus* Makino with their in vitro and in silico studies target anti-inflammation by suppressing ERK 1/2 MAPK signaling

Duc Dat Le[a,†], Thinhulinh Dang[a,†], Seok-Geun Lee[b,c], Vinhquang Trương[a], and Mina Lee[a,d*]

[a] College of Pharmacy and and Research Institute of Life and Pharmaceutical Sciences, Sunchon National University, 255 Jungangno, Suncheon, Jeonnam, 57922, Republic of Korea

[b] Graduate School, Kyung Hee University, Seoul, 02447, Republic of Korea

[c] BioNanocomposite Research Center, Kyung Hee University, Seoul, 02447, Republic of Korea

[d] Department of Natural Cosmetics Science Smart Beautytech Research Institute, Sunchon National University, 255 Jungangno, Suncheon, Jeonnam 57922, Republic of Korea

[†] Authors equally contribute to this work

[*] Correspondence: minalee@scnu.ac.kr; Tel.: 82-61-750-3764; Fax: 82-61-750-3708

Spectroscopic data of syringin (**1**)

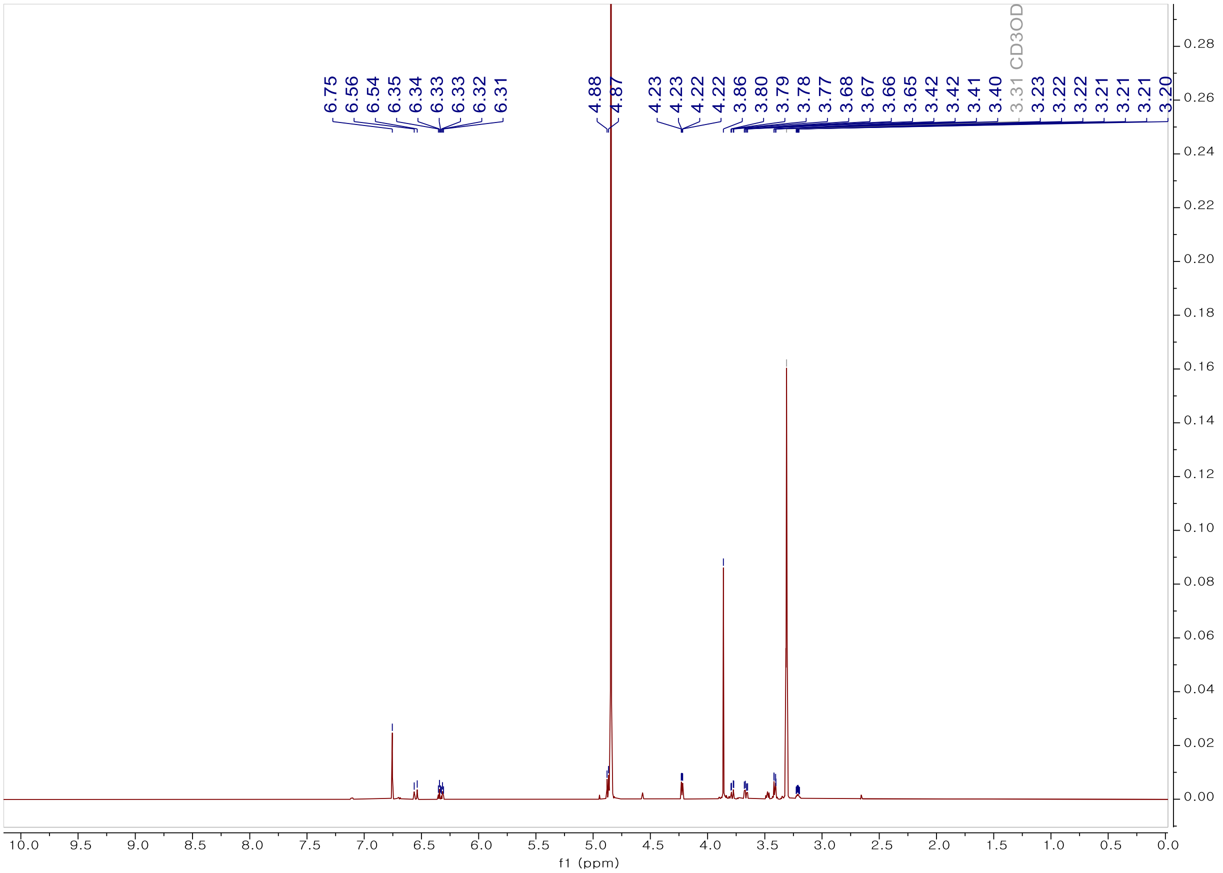

Figure S1. ^{1}H NMR (600 MHz, CD_3OD) spectrum of isolated compound **1**.

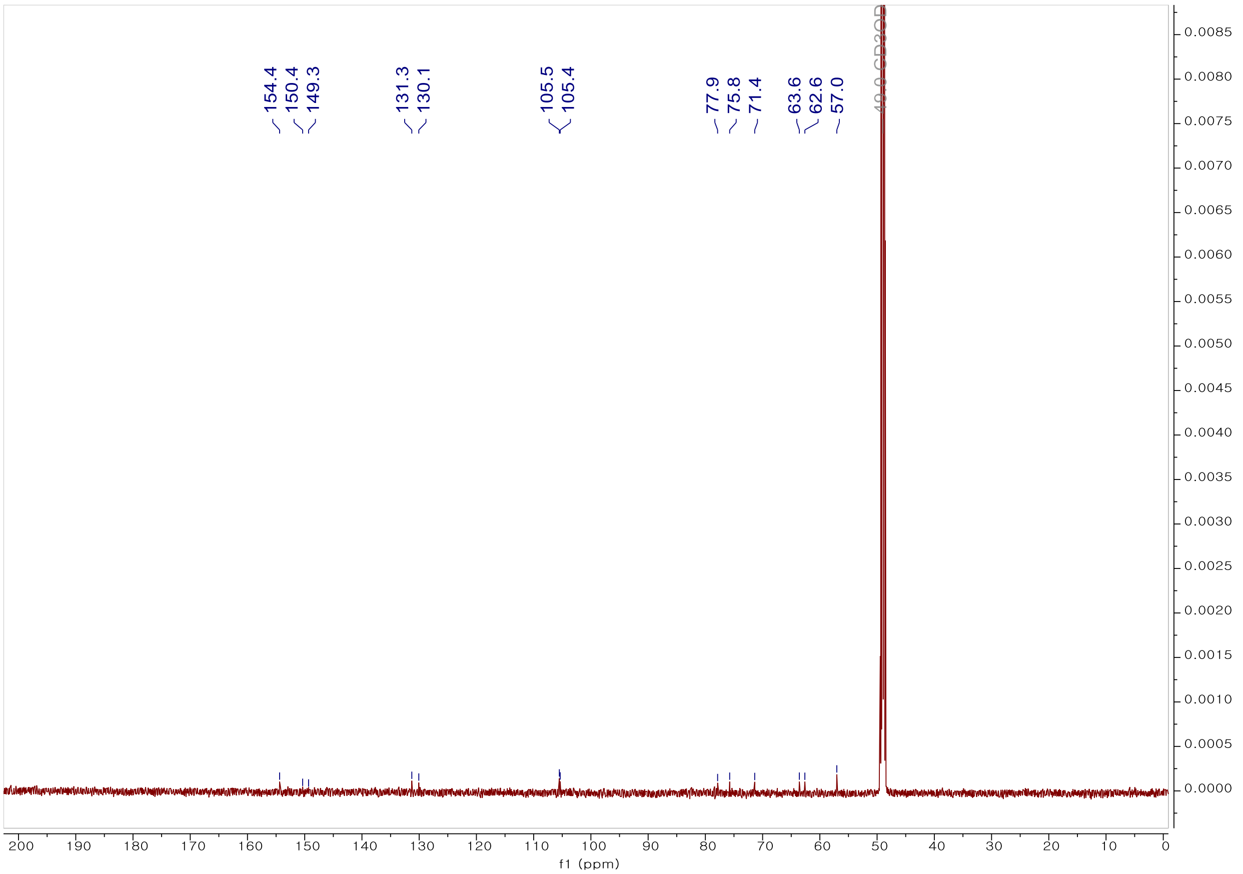

Figure S2. ^{13}C NMR (150 MHz, CD_3OD) spectrum of compound **1**.

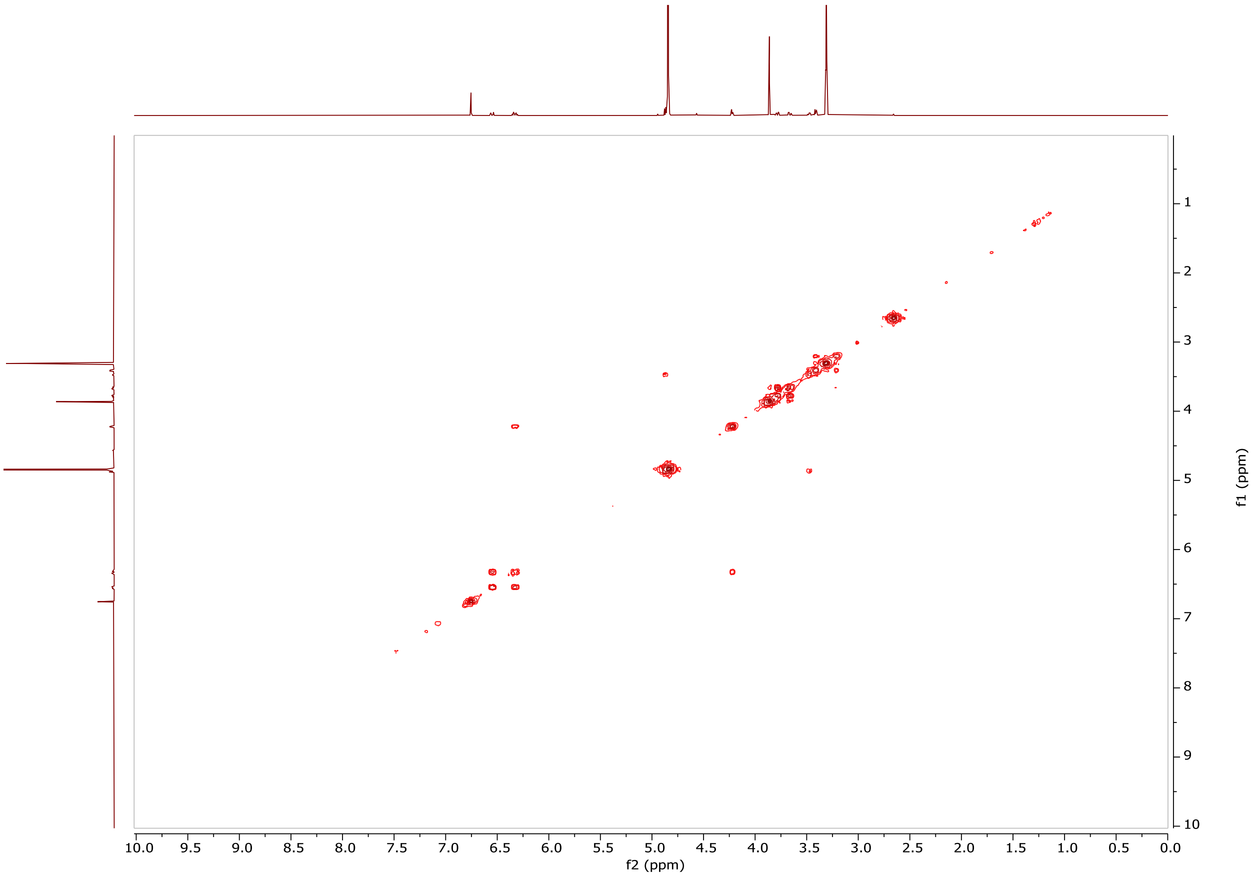

Figure S3. ^1H–^1H COSY spectrum of compound **1**.

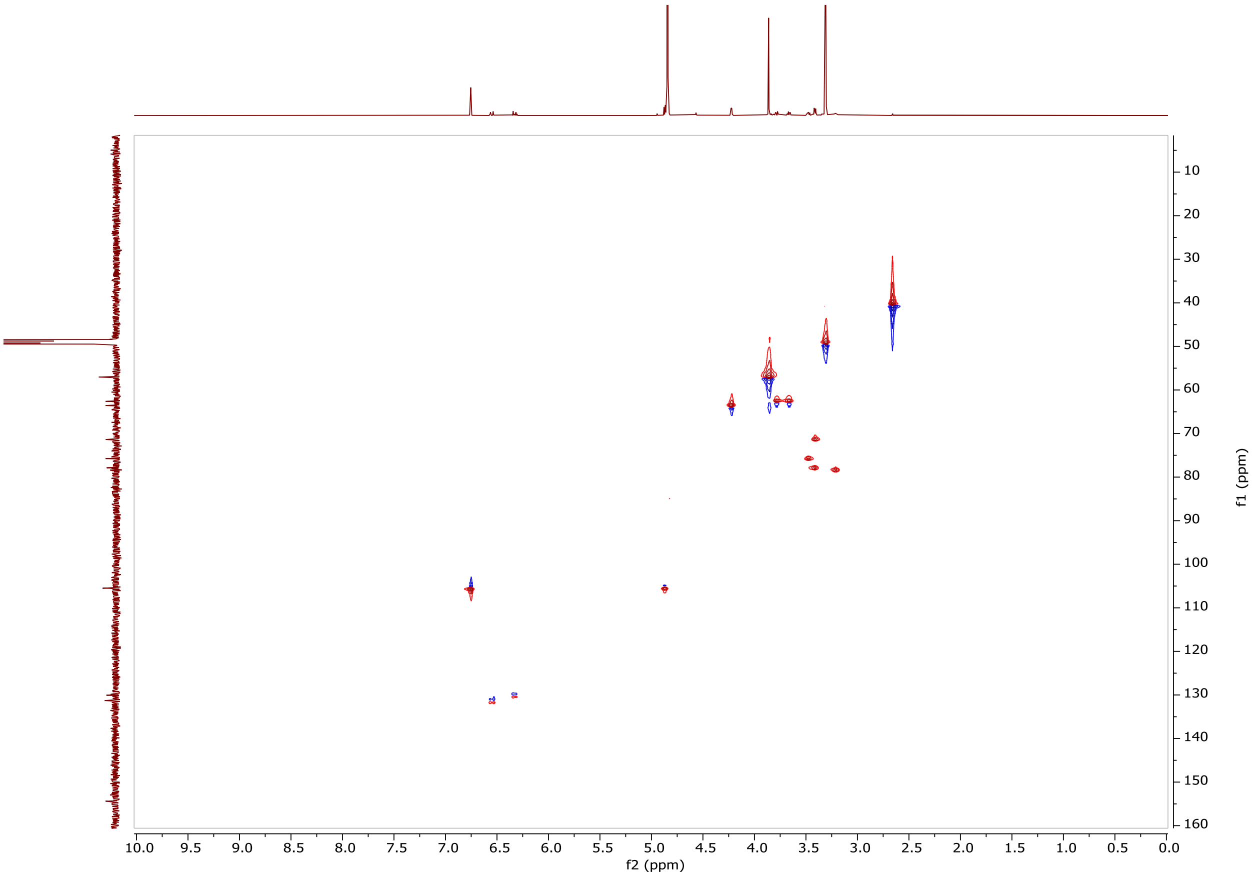

Figure S4. ^{1}H–^{13}C HSQC spectrum of compound **1**.

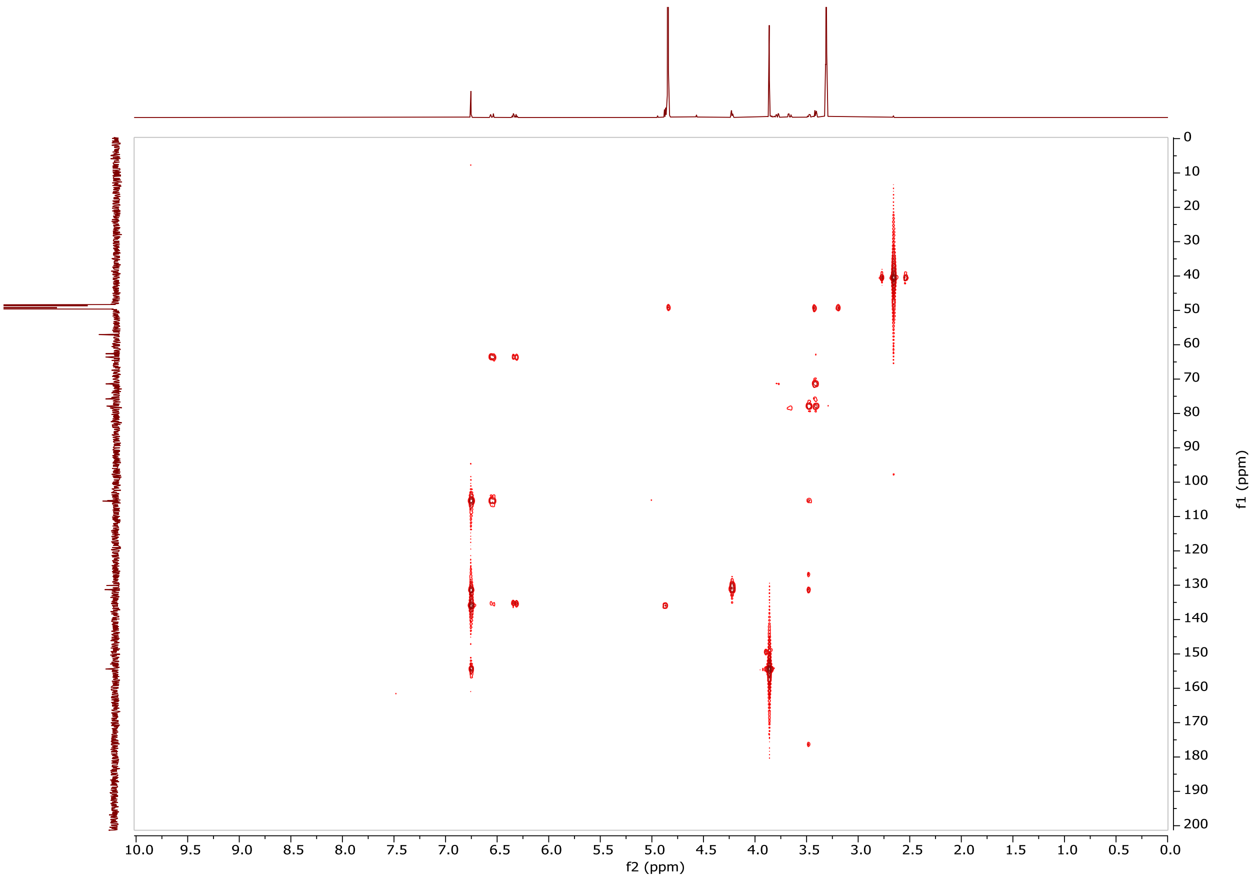

Figure S5. ^{1}H–^{13}C HMBC spectrum of compound **1**.

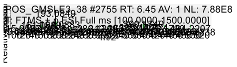

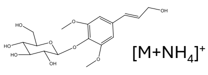

$[M+NH_4]^+$

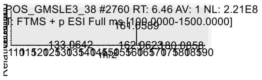

Figure S6. HRESIMS spectrum of compound **1** detected in positive ion mode.

Spectroscopic data of 8-epikingiside (**4**)

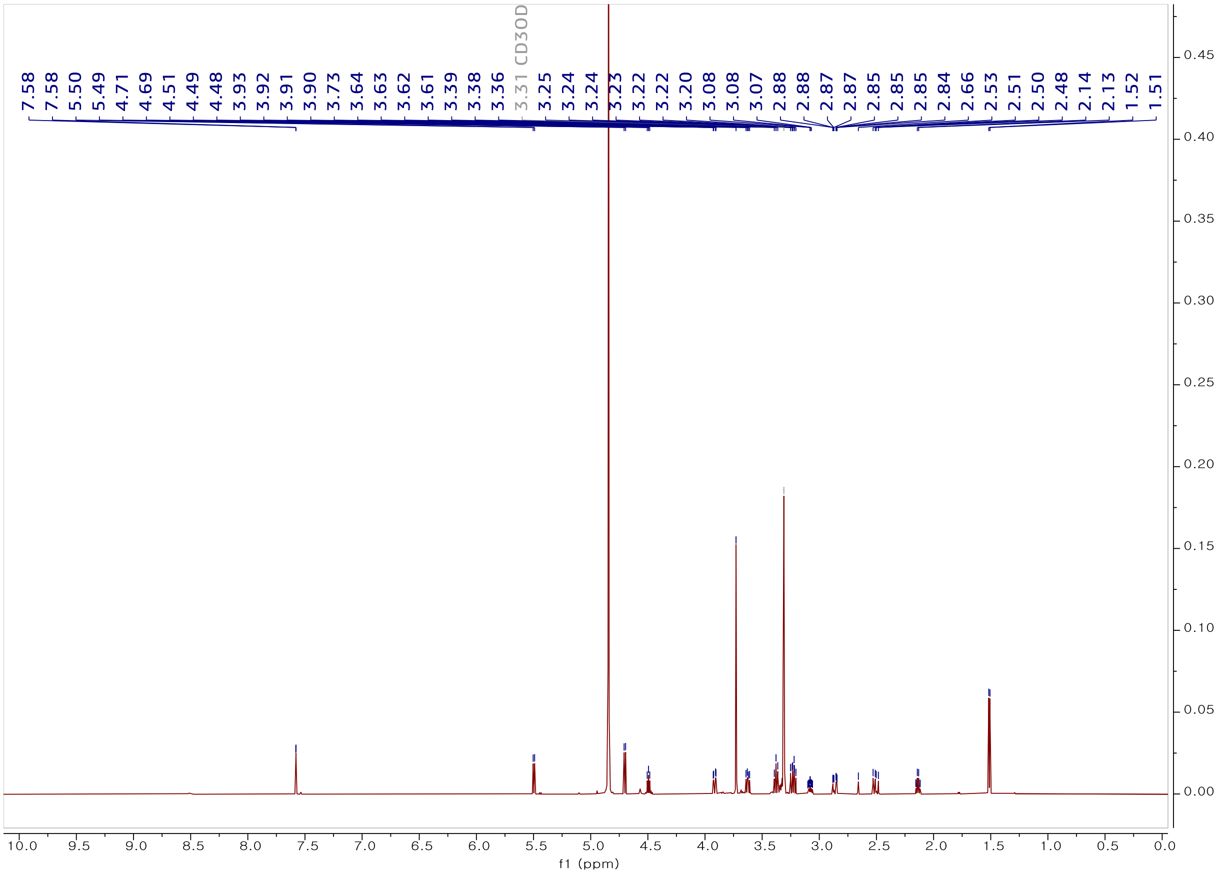

Figure S7. ^{1}H NMR (600 MHz, CD_3OD) spectrum of compound **4**.

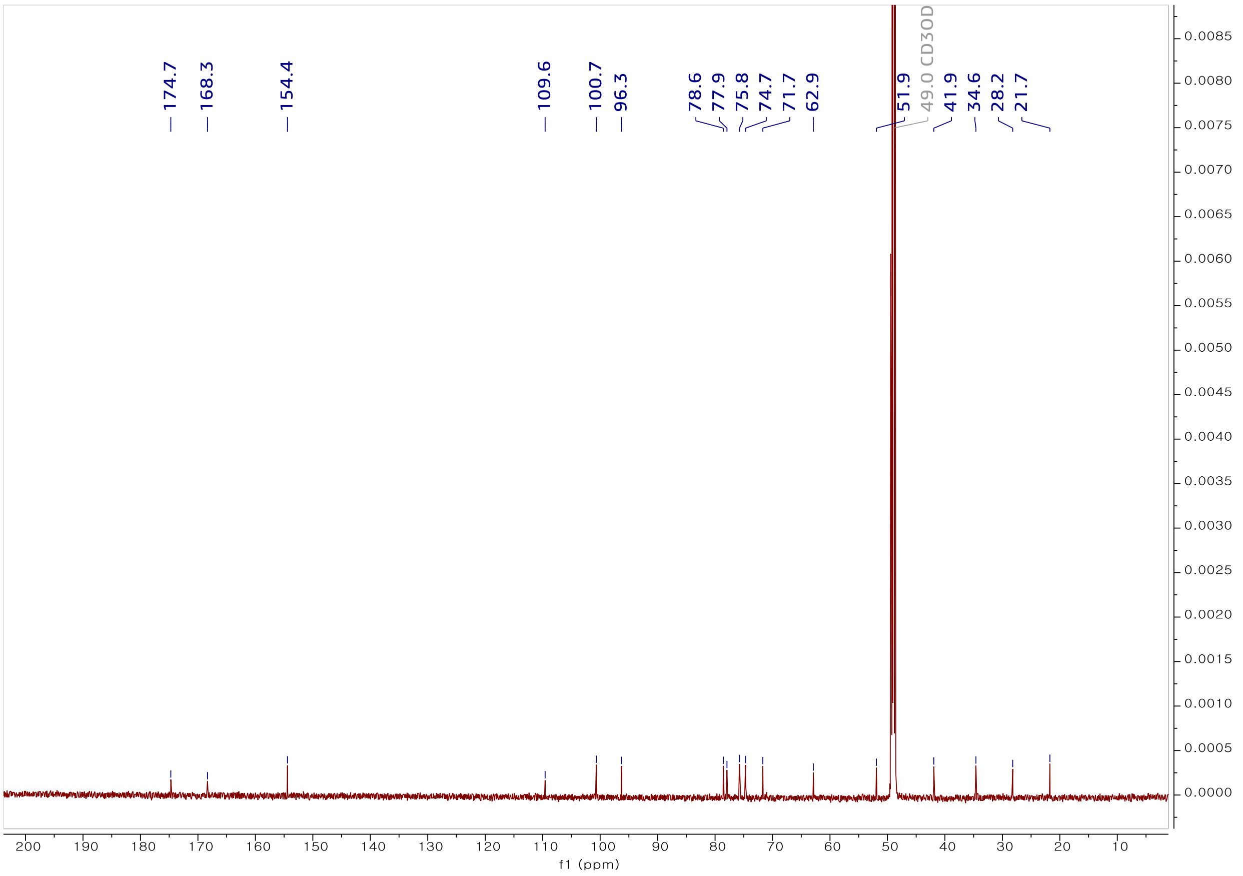

Figure S8. ^{13}C NMR (150 MHz, CD_3OD) spectrum of compound **4**.

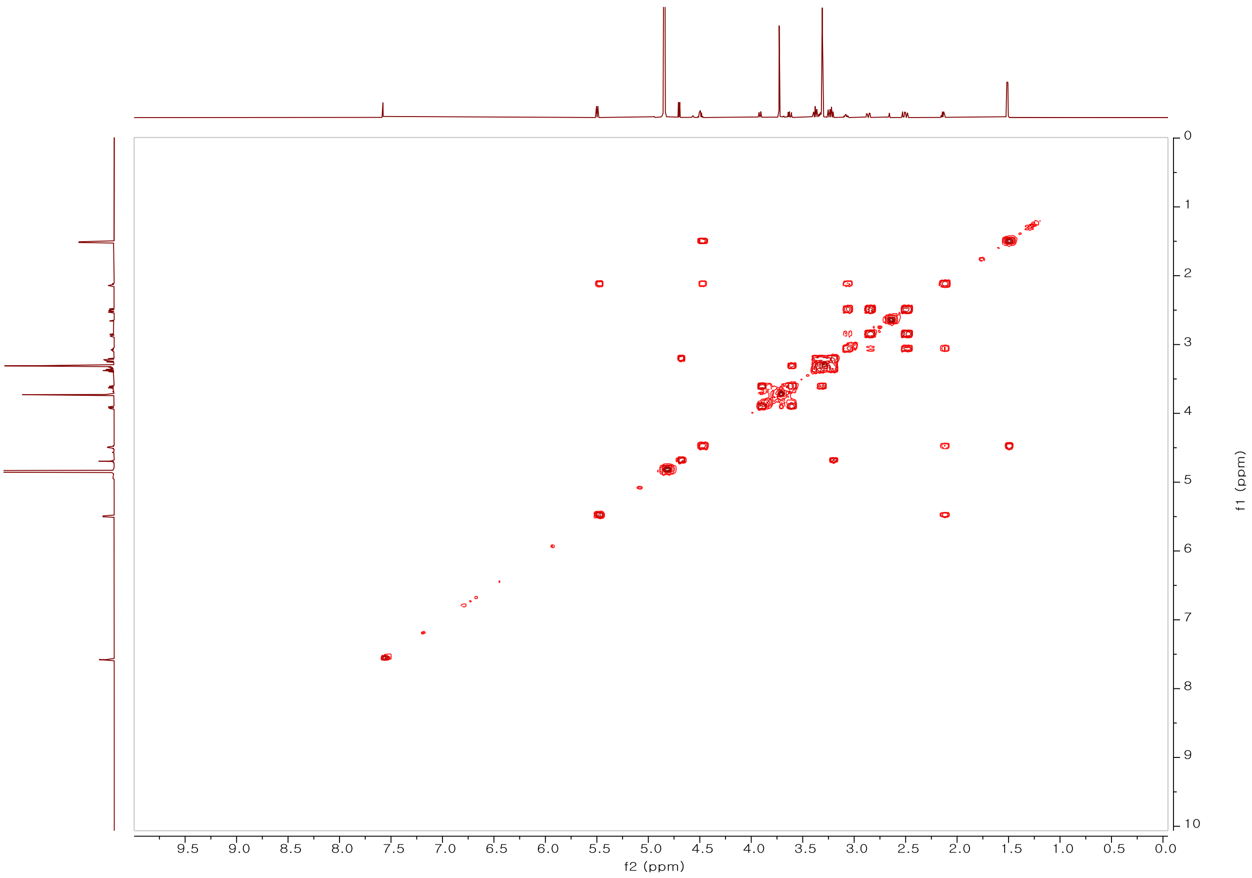

Figure S9. ^{1}H–^{1}H COSY spectrum of compound **4**.

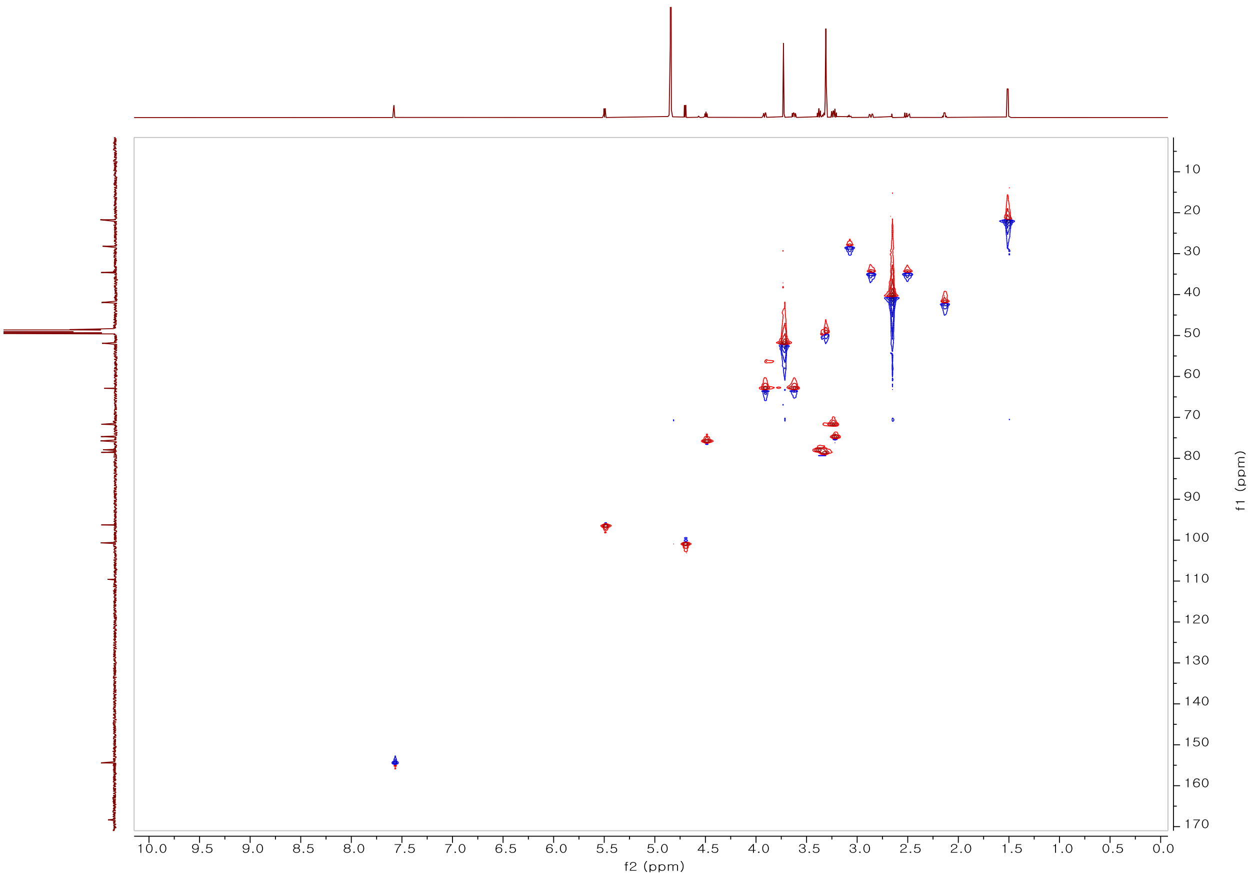

Figure S10. ^{1}H–^{13}C HSQC spectrum of compound **4**.

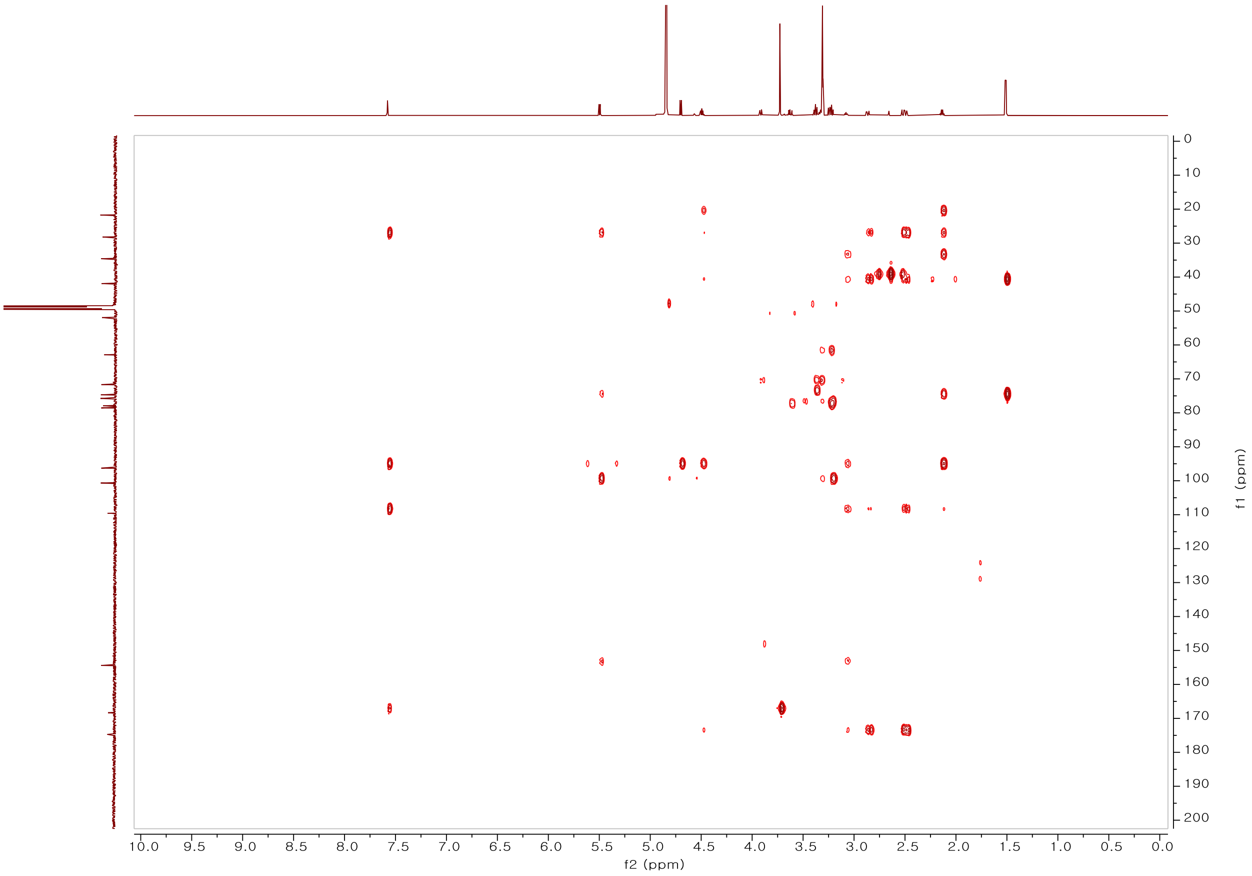

Figure S11. ^{1}H–^{13}C HMBC spectrum of compound **4**.

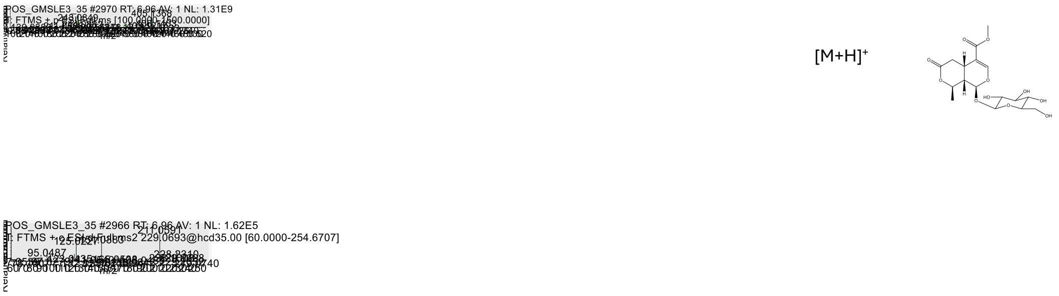

Figure S12. HR-ESI-MS/MS spectrum of compound **4** detected in positive ion mode.

Spectroscopic data of fragrans D1 (**9**)

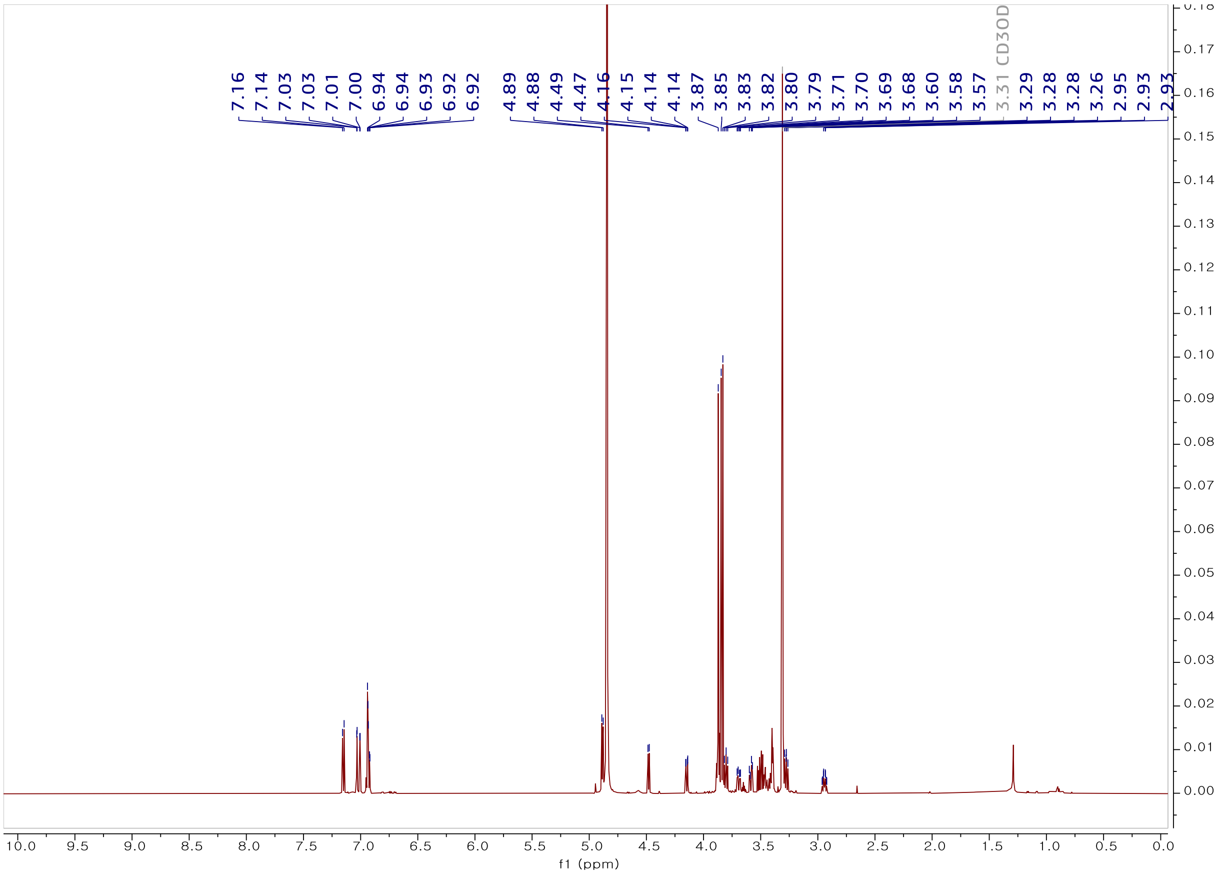

Figure S13. ^{1}H NMR (600 MHz, CD_3OD) spectrum of compound **9**.

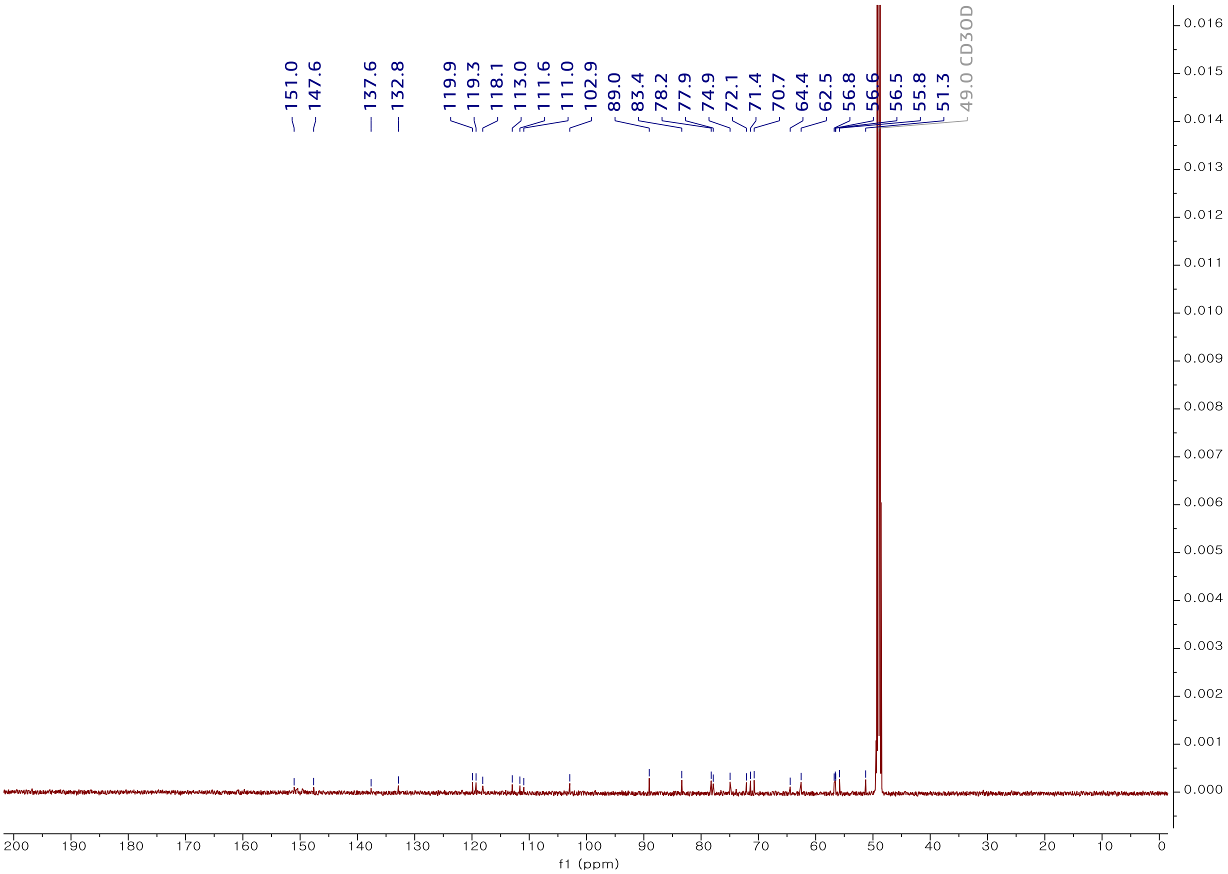

Figure S14. ^{13}C NMR (150 MHz, CD_3OD) spectrum of compound **9**.

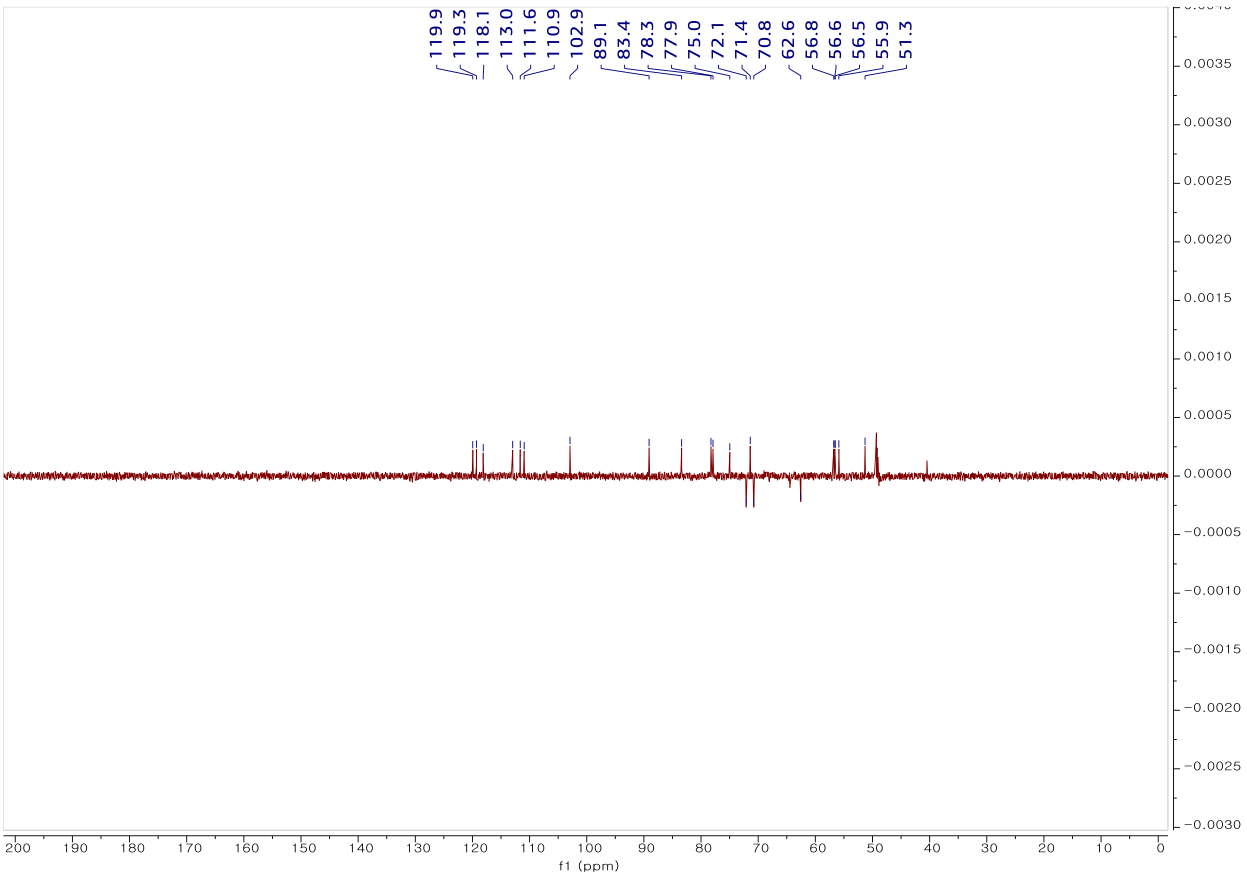

Figure S15. DEPT spectrum of compound **9**.

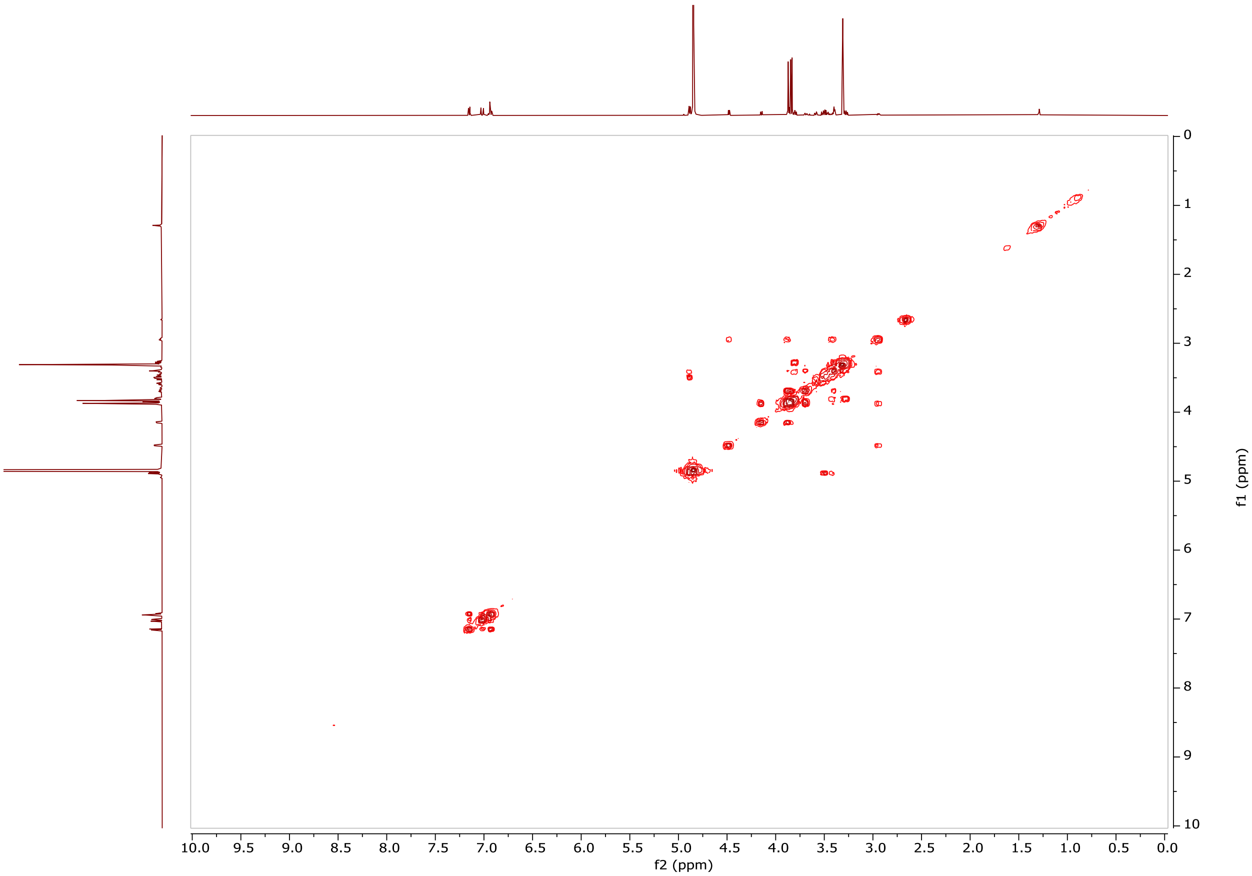

Figure S16. ^{1}H–^{1}H COSY spectrum of compound **9**.

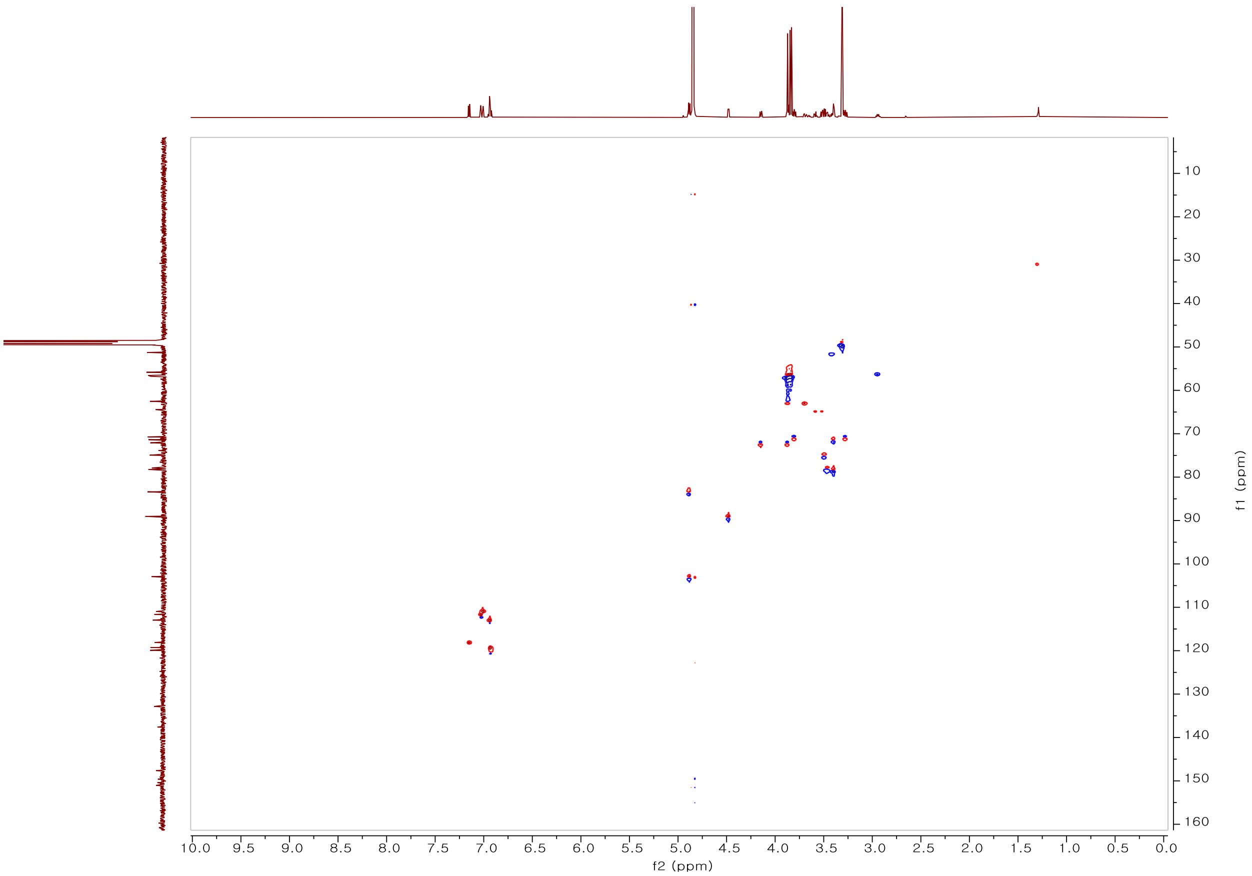

Figure S17. ^{1}H–^{13}C HSQC spectrum of compound **9**.

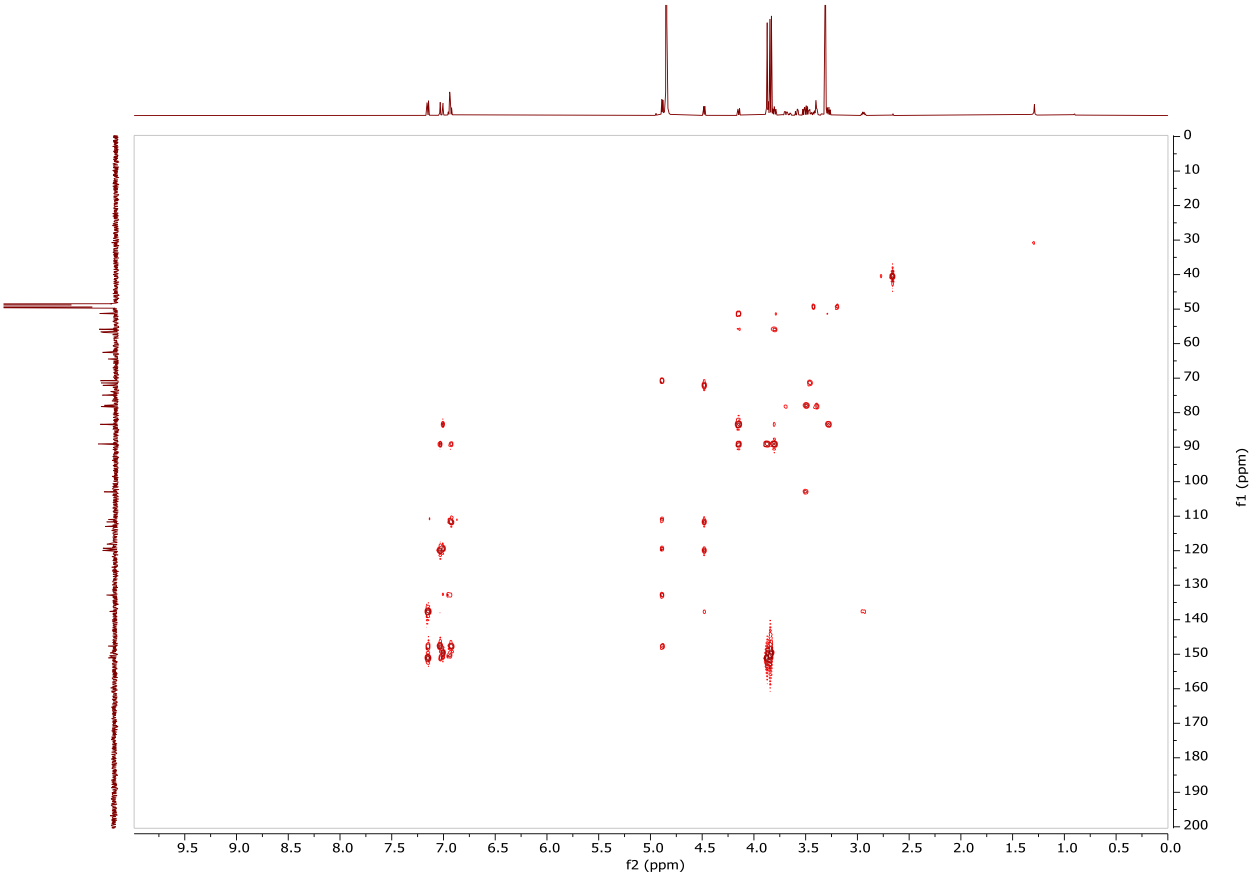

Figure S18. ^{1}H–^{13}C HMBC spectrum of compound **9**.

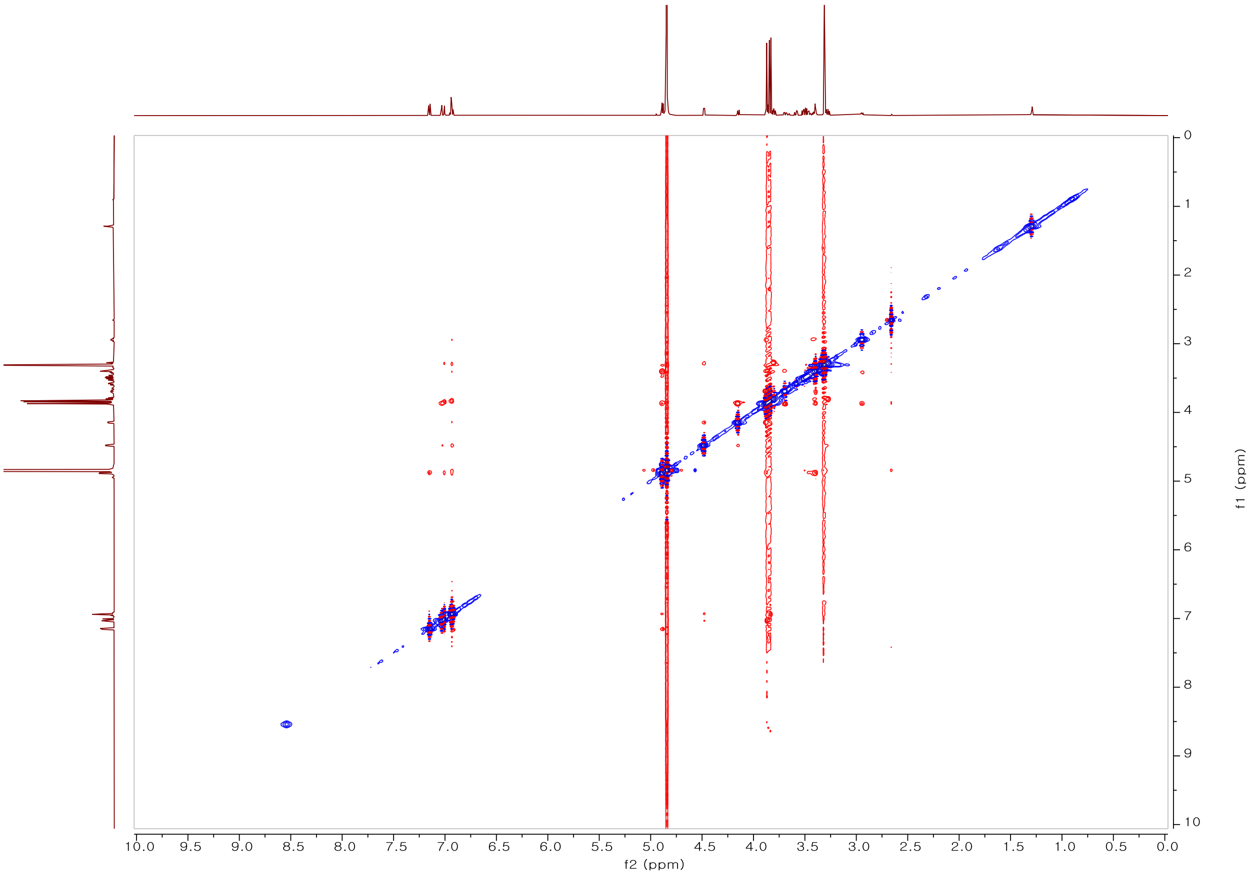

Figure S19. ^{1}H–^{1}H NOESY spectrum of compound **9**.

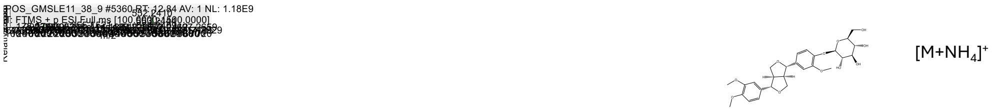

Figure S20. HRESIMS spectrum of compound **9** detected in positive ion mode.

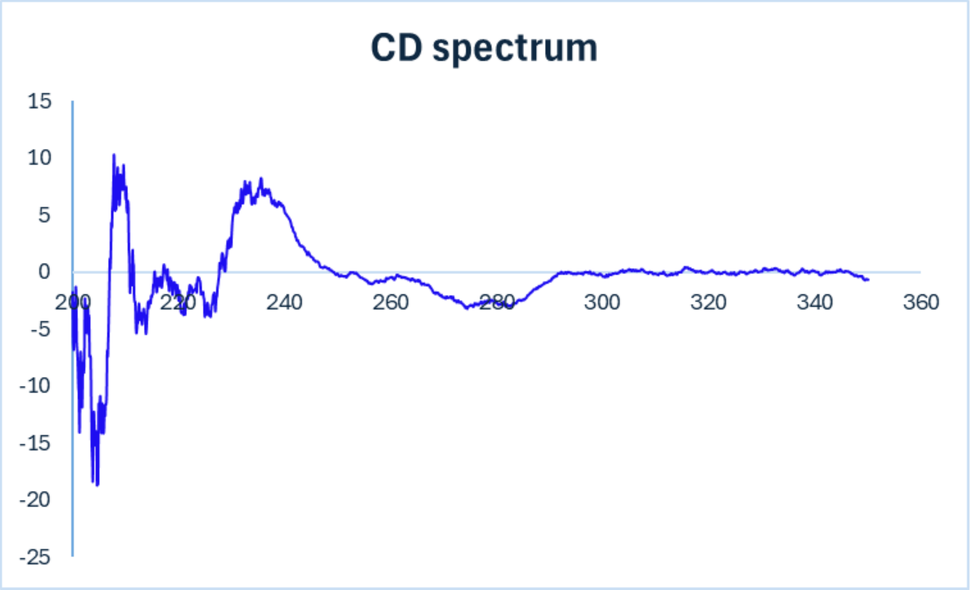

Figure S21. CD spectrum of compound **9**.
